# Antidiabetic potential of fenugreek (*Trigonella foenum‐graecum*): A magic herb for diabetes mellitus

**DOI:** 10.1002/fsn3.4440

**Published:** 2024-09-05

**Authors:** Dipto Kumer Sarker, Pallobi Ray, Ashit Kumar Dutta, Razina Rouf, Shaikh Jamal Uddin

**Affiliations:** ^1^ Pharmacy Discipline, Life Science School Khulna University Khulna Bangladesh; ^2^ East Tennessee State University Johnson City Tennessee USA; ^3^ Department of Pharmacy, Faculty of Life Science Bangabandhu Sheikh Mujibur Rahman Science & Technology University Gopalganj Bangladesh

**Keywords:** bioactive compounds, diabetes, fenugreek, molecular mechanism, *Trigonella foenum‐graecum*

## Abstract

Fenugreek (*Trigonella foenum‐graecum*) is a widely grown dietary herb in Asia, and its seeds are traditionally used for several diseases, including diabetes. The seeds and leaves possess a variety of compounds that play an important role in regulating their hypoglycemic effect. However, so far, no extensive systematic review exists on its antidiabetic effect, highlighting the molecular mechanisms and isolated compounds. The purpose of this review is to summarize the preclinical and clinical antidiabetic properties of fenugreek and its isolated compounds by focusing on underlying mechanisms. PubMed, Google Scholar, Science Direct, and Scopus databases were searched to retrieve articles until June, 2024. Preclinical studies demonstrated that the antidiabetic effect of fenugreek was mostly associated with enhanced glucose transporter type‐4 (GLUT4) translocation and hexokinase activity, decreased glucose‐6‐phosphatase and fructose‐1,6‐bisphosphatase activities, inhibited α‐amylase and maltase activities, protected β cells, and increased insulin release. Furthermore, few studies have reported its role as a glucagon‐like peptide‐1 (GLP‐1) modulator, 5′‐AMP‐activated kinase (AMPK) activator, and dipeptidyl peptidase‐IV (DPP‐IV) inhibitor. Further clinical trials showed that fenugreek seeds improved blood glucose levels, insulin resistance, insulin sensitivity, and lipid profiles. This study highlights significant evidence of the antidiabetic effect of fenugreek and its isolated compounds; therefore, it could be a potential therapy for diabetes.

## Graphical abstract

This systematic review has been conducted on 98 preclinical and 24 clinical antidiabetic studies of fenugreek and its isolated compounds. We have found significant antidiabetic effect of fenugreek that is primarily a consequence of activation of the PI3K/Akt pathway, restoration of glucose homeostasis, and protection of the β cells. The clinical studies further revealed that fenugreek reduced blood glucose and insulin resistance and enhanced insulin sensitivity.

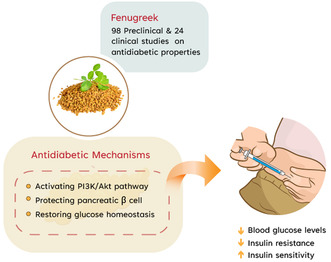

## 1 INTRODUCTION

Diabetes mellitus (DM) is one of the most prevalent diseases in today's world, and it has now become a serious public health problem (Antar et al., 2023). According to the International Federation of Diabetes (IDF) Diabetes Atlas 2021, 537 million people worldwide have diabetes, with the number expected to rise to 643 and 783 million by 2030 and 2045, respectively. In recent years, Bangladesh has seen a large number of diabetic people, with an estimated 13.1 million in 2021 and a predicted 22.3 million by 2045 (Magliano & Boyko, 2022).

Diabetes mellitus is a metabolic disorder characterized by chronic hyperglycemia resulting from impairment of insulin secretion, action, or both (Antar et al., 2023). Type 1 and type 2 are the two major forms of DM. Type 1 diabetes is caused by the autoimmune destruction of cells, resulting in a complete deficiency of insulin secretion (Skamagas et al., 2008). On the other hand, type 2 diabetes develops due to insulin resistance and β‐cell dysfunction (Li, Lu, et al., 2018). Among all types of DM, type 2 diabetes is the most common (90%) (Dahlén et al., 2021). However, long‐term diabetes is a significant contributor to micro‐ and macrovascular complications that cause nephropathy, retinopathy, neuropathy, cardiovascular diseases, peripheral artery diseases, and stroke (Dahlén et al., 2021). Although tight glycemic control minimizes these complications, the morbidity rate linked to them keeps increasing (Chawla et al., 2016). Since there is currently no cure for DM, the development of new therapies for controlling diabetes has become a vital approach (Dahlén et al., 2021).

Plants have been widely used for different diseases since ancient times (Chaachouay & Zidane, 2024). The phytochemical compounds present in plant extracts are responsible for their therapeutic effects, including managing diabetes, making them a rich source of new drugs (Abdelghffar et al., 2022). Fenugreek (*Trigonella foenum‐graecum*), belonging to the Fabaceae family, has been reported to have antidiabetic (Li, Lu, et al., 2018), antioxidant (Pandey & Awasthi, 2015), antihyperlipidemic (Saxena & Saxena, 2009), anti‐inflammatory (Pournamdari et al., 2018), antiobesity (Nagulapalli Venkata et al., 2017), and miscellaneous pharmacological effects. In Asia and Africa, its seeds are traditionally used to manage diabetes (Ahmad et al., 2016). Numerous preclinical and clinical studies have been conducted so far to examine its antidiabetic benefits (Geberemeskel et al., 2019; Hota et al., 2023). Bioactive substances present in seeds, such as galactomannan, (2*S*,3*R*,4*S*) 4‐hydroxyisoleucine, saponin, diosgenin, trigonelline, quercetin, orientin, vitexin, and isovitexin, have been shown to reduce hyperglycemia (Hamden et al., 2013; Kamble et al., 2013; Li, Lu, et al., 2018; Rawat et al., 2014; Saravanan et al., 2014; Zhang et al., 2020). The leaves, on the other hand, are rarely used but contain saponins, quercetin, catechin, cinnamic acid, coumaric acid, and soluble fibers (Wani & Kumar, 2018). Considering the traditional uses and vast antidiabetic studies, this study aims to review the antidiabetic activity of fenugreek, focusing on their insights into reducing hyperglycemia, so that fenugreek can be utilized more effectively for managing diabetes.

## 2 METHODOLOGY

### 2.1 Literature search strategy

The literature search was conducted in PubMed, Google Scholar, Science Direct, and Scopus using the term “Fenugreek” with “Diabetes Mellitus,” “Diabetes,” and “Hyperglycemia.” In this study, we focused on the literature only in English due to the language barrier and efficiency published until June, 2024. Several inclusion criteria were used, including (a) in vitro studies, (b) in vivo studies, (c) clinical trials, and (d) antidiabetic studies of the compounds isolated from fenugreek. Key data such as the surname of the first author, year of publication, fenugreek and its isolated compounds, test model, observations, results, concentration tasted, and molecular mechanisms were carefully extracted from the included articles. Articles that did not fulfill the above criteria and did not report any antidiabetic effect of fenugreek or its isolated compounds were excluded from the study.

## 3 RESULTS AND DISCUSSION

**FIGURE 1:**
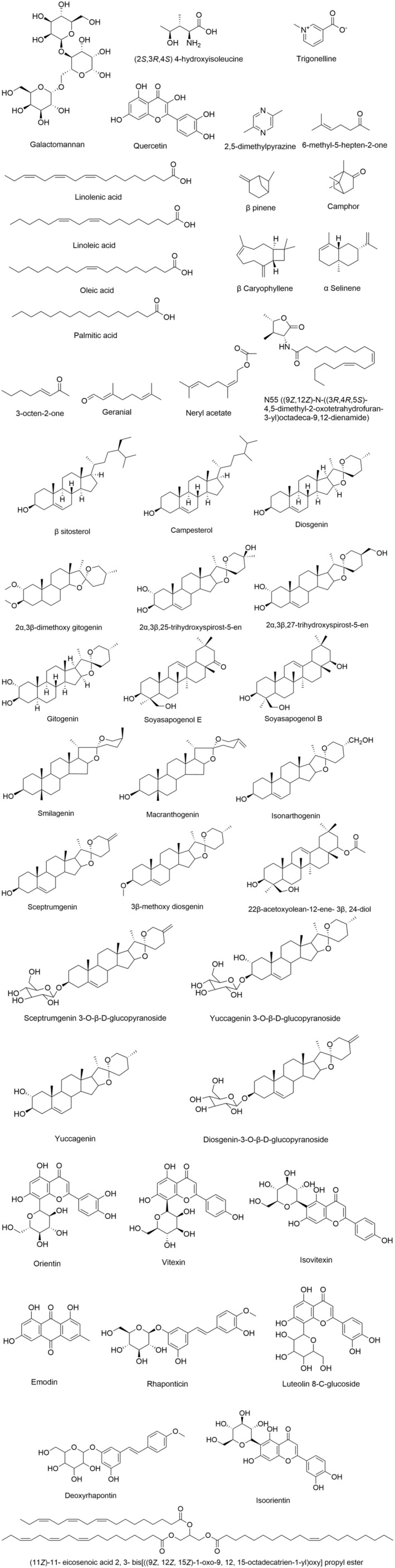
Chemical structures of compounds isolated from fenugreek seeds.

In this study, a total of 98 articles were found reporting the antidiabetic effect of fenugreek in either in vitro, in vivo, or clinical trials. The majority of these studies used fenugreek seeds or its isolated compounds rather than its leaves. Table 1 indicates the in vitro and in vivo studies of fenugreek and its isolated compounds. Figure 1 represents the chemical structures of reported compounds isolated from fenugreek seeds.

**TABLE 1:** Preclinical (in vivo and in vitro) studies of fenugreek and its isolated compounds.

(A) In vitro studies of fenugreek						
Test model	Parts	Dose/concentration	Route of administration	Mechanism of action	Formulation	Ref.
α‐amylase and α‐glucosidase activity; primary human subcutaneous adipocytes cell	Seeds	25–30 μL; 3–11 μg/mL	In vitro; Cell line	↓ α‐amylase; ↑ PDK1 and GLUT4 expression	Ethanol extract	Kan et al. (2017)
α‐glucosidase activity	Seeds	20 μL	In vitro	↓ α‐glucosidase activity	Ethanol extract	Zhang et al. (2020)
Simulated small intestinal digesta	Gum	0.43–1.3% (w/v)	In vitro	↓ amylolysis	Water‐extracted fenugreek gum	Repin et al. (2017)
3 T3‐L1 cells	Seeds	1000 mg/mL	Cell line	Enhancement of glucose transport	SDF fraction	Hamden et al. (2017)
α‐amylase activity	Seeds	0.4 mg/mL	In vitro	↓ α‐amylase activity	Aqueous extract	Gad et al. (2006)
Rat pancreatic INS‐1β‐cells; mouse 3 T3‐L1 adipocytes	Seeds	25–1000 μg/mL	Cell line	↓ Intestinal absorption; ↑ consumption of glucose by adipocytes	Aqueous extract	Kaur et al. (2011)
Rat pancreas; rat islets and human pancreas	Seeds	100–1000 μM	Cell line	↑ Insulin secretion through a direct action on β‐cells	Ethanol extract	Sauvaire et al. (1998)
Rabbit intestinal brush border membrane vesicle; Isolated hepatocyte suspension	Seeds	0.3–1.65 mg/mL	In vitro	↓ SGLT‐1 mediated glucose absorption; ↓ glucagon induced HGPa activity	Lipid extract; crude ethanolic extract; saponin‐free; saponin; sapogenin; gum fiber fraction	Al‐Habori et al. (2001)
CHO‐HIRc‐mycGLUT4eGFP; 3 T3‐L1‐mycGLUT4; 3 T3‐L1 adipocytes and HepG2 cells	Seeds	Different concentration	Cell line	↑ Translocation of GLUT4; ↑ Tyrosine phosphorylation of IR, IRS‐1 and p85 subunit of PI3K	Dialyzed aqueous extract	Vijayakumar et al. (2005)
Fluorometric method	Seeds	Different concentration	In vitro	↓ DPP‐IV	Hot water extract	Ansari et al. (2021)
Fatty acid (palmitate) induced insulin resistance in L6 myotubes	Seeds	25 μM	Cell line	Restoration of altered IRS1/AKT/AS160/GSK pathway; ↑ IRS‐1 tyrosine phosphorylation	Undefined	Maurya et al. (2014)
3 T3‐L1 adipocytes	Seeds	Different concentration	Cell line	↑ Activation of PKB and AMPK	*n*‐Butanol extracts	Li, Luan, et al. (2018)
Lysed diabetic human whole blood	Seeds	25–200 μg	In vitro	Undefined	Chloroform extract	Bansode, Gupta, et al. (2017)
Pancreatic ductal stem cell culture	Seeds	100–800 μL of 5 mM concentration	Cell line	Regeneration of pancreatic β cells; adipogenesis	Aqueous alcohol extract	Shah et al. (2009)
Nonenzymatic glycosylation of hemoglobin assay	Seeds	1 mL	In vitro	Undefined	Methanol extract	Devi and Raju (2020)
L6 myotubes	Seeds	10 μM	Cell line	↑ AMPK; upregulation of the PGC‐1α, PGC‐1β, CPT 1 and CPT 2 genes	Alcohol extract	Rawat et al. (2014)
α‐amylase and α‐glucosidase activity	Seeds	1–9% w/v	In vitro	↓ α‐amylase and α‐glucosidase	Boiled and unboiled extract	Arooj et al. (2024)

(B) In vivo antidiabetic studies of fenugreek						
Test model	Parts	Dose/concentration	Route of administration	Mechanism of action	Formulation	Ref.
STZ‐induced diabetes in rat model	Seed oil	3 g/kg	Oral	Undefined	Oil	Parveen et al. (2019)
STZ‐induced diabetes in rat model	Seeds	10%	Oral	↑ Insulin secretion; ↑ insulin sensitivity; regeneration of β cells	Powder	Pradeep and Srinivasan (2017)
STZ‐induced diabetes in rat model	Seeds	0.05 g/mL	Oral	Protection of β cells; ↓ Insulin resistance; regulation of gluconeogenesis	Ethanol extract	Jiang et al. (2018)
STZ‐induced diabetes in rat model	Seeds	100 mg/kg	Undefined	Undefined	Ethanolic extract	Hosseini et al. (2020)
STZ‐induced diabetes in rat model	Seeds	1.74–0.87 mg/kg	Oral	Undefined	Aqueous extract	Haghani et al. (2016)
STZ‐induced diabetes in rat model	Seeds	0.5 g/kg	Oral	↓ Carbohydrate digestion and absorption; ↑ peripheral insulin action	Methanol extract‐free residue of SDF	Hannan et al. (2007)
STZ‐induced diabetes in rat model	Leaves	0.5, 1 g//kg	Oral	↑ HK, ↓G6Pase, FBPase in liver and kidney; ↑ β‐cells; reactivation of GS	Powder	Devi et al. (2003)
Starch‐fed diet and STZ‐induced diabetes in rat model	Seed mucilage	25 g	Oral	Restoration of intestinal and renal maltase enzyme activity	Water extract	Kumar et al. (2005)
High sucrose diet fed and STZ‐induced diabetes in rat model	Seeds	50 mg/kg	Undefined	Improvement of mitochondrial biogenesis	Alcohol extract	Rawat et al. (2014)
STZ‐induced diabetes in rat model	Seeds	0.87–1.74 g/kg	Oral	Undefined	Water boiled extract	Arshadi, Bakhtiyari, et al. (2015)
STZ‐induced diabetes in rat model	Seeds	1 g/kg	Oral	Improving hyperlipidemia; ↑antioxidant activity	Powder suspended in water	Marzouk et al. (2013)
HFD and STZ‐induced diabetes in rat model	Seeds	150–450 mg/kg	Oral	Undefined	Water‐soluble seed extract	Swaroop et al. (2014)
STZ‐induced diabetes in rat model	Seeds	1.5 g/kg	Oral	↑ Hepatic glycogen storage, glucose metabolism through HMP; ↓ hepatic gluconeogenesis, starch digestion and absorption	Aqueous extract	Gad et al. (2006)
STZ‐induced diabetes in rat model	Seeds	0.8–1.6 g/kg	Oral	↑ GLUT 4 signaling pathways	Aqueous extract	Arshadi, Ali Azarbayjani, et al. (2015)
STZ‐induced diabetes in rat model	Seeds	2 g/kg	Oral	↑ GK, HK, PFK, glycogen content	Defatted extract	Vats et al. (2003)
STZ‐induced diabetes in rat model	Seeds	20%	Oral	↓ Intestinal glucosidase	Pellets	Riyad et al. (1988)
STZ‐induced diabetes in rat model	Seeds	0.5 g/kg	Oral	↓Carbohydrate and fat absorption	SDF fraction	Hannan et al. (2007)
STZ‐induced diabetes in rat model	Seeds	500–750 mg/kg	Oral	Undefined	Hydroalcoholic extract	Manik et al. (2013)
STZ‐induced diabetes in rat model	Seeds	40 mg/mL	Oral	↑ HK, G6PD, ↓ G6Pase activities; sensitization of β‐cells or regeneration the β‐cells	Aqueous extract	Bera et al. (2013)
STZ‐induced diabetes in rat model	Seeds	0.44–1.74 g/kg	Oral	Undefined	Aqueous extract	Xue et al. (2007)
STZ‐induced diabetes in rat model	Seeds	8%	Oral	↓Maltase, lactase, and sucrase activities, ↓ intestinal lipase activity	Ethanol extract	Hamden, Jaouadi, et al. (2010)
STZ‐induced hyperglycemic rats	Seeds	50 mg/kg	Oral	Presence of alkaloid; ↓ blood glucose, lipid profile to normal and oxidative stress	Alkaloid extract	Abou El‐Soud et al. (2007)
STZ‐induced diabetes in rat model	Seeds	1 g/kg	Oral	Undefined	Ethanol extract	Jyothi et al. (2017)
STZ‐induced diabetes in rat model	Seeds	1–2 mg/mL	Oral	↑ Activation of 6PFK1 activity in intestinal and liver cells	Water extract	Ali et al. (2013)
STZ‐induced diabetes in rat model	Seeds	100 mg/kg	IP and oral	Undefined	Undefined	Baset et al. (2020)
STZ‐induced diabetes in rat model	Seeds	300 mg/kg	Oral	Undefined	Aqueous extract	Al‐Chalabi et al. (2019)
STZ‐induced diabetic rats	Seeds	200–400 mg/kg	Oral	Undefined	Methanol extract	Devi and Raju (2020)
Nitrate‐induced diabetes in rat model	Seeds	5%	Oral	Undefined	Powdered seeds	El‐Wakf et al. (2015)
Alloxan‐induced diabetes in rat model	Seeds	10%	Oral	Prevention of β‐cell destruction; ↑ insulin secretion; ↓ lipid peroxidation	Oil	Hamden, Masmoudi, et al. (2010)
Alloxan‐induced diabetes in rat model	Seeds	5%	Oral	↓ α‐amylase and maltase in both pancreas and plasma; restoration of β‐cell structure	Omega‐3 fatty acid‐rich fenugreek essential oil; fenugreek essential oil	Hamden et al. (2011)
Alloxan‐induced diabetes in rabbit model	Seeds	25–175 mg/kg	Oral	Undefined	Water extract	Moorthy et al. (2010b)
Alloxan‐induced diabetes in rat model	Seeds	2 g/kg	Oral	Undefined	Grinded seeds	Saadh (2020)
Alloxan‐induced diabetes in rat model	Seeds	0.1–2 g/kg	Oral	Undefined	Ethanol extract	Mowl et al. (2009)
Alloxan‐induced diabetes in rat model	Seeds oil	50 mg/kg	Oral	↑ Insulin sensitivity; ↓ α amylase, lipase activity; restoration of ACE activity	Hexane extract	Hamden et al. (2017)
Alloxan‐induced diabetes in rat model	Seeds	100 mg/kg	Oral	Due to free radical scavenging properties	Ethanolic extract	Yella et al. (2019)
Alloxan‐induced diabetes in BALB/cJ mice model	Seeds	15 mg/kg	Intraperitoneal	↑ Liver GK, HK activity	Dialyzed aqueous extract	Vijayakumar and Bhat (2008)
Alloxan‐induced subdiabetic, mild diabetic, and severely diabetic rabbits	Seeds	50 mg//kg	Oral	↑ Serum insulin and sensitivity of tissues to insulin action	Water extract	Puri et al. (2011)
Alloxan‐induced diabetes in rat model	Seeds	100 mg/kg	Oral	Improve liver glycogen and body weight	Chloroform extract (Saponin fraction)	Bansode, Salalkar, et al. (2017)
Alloxan‐induced diabetes in mice model	Seeds	1–15 mg/kg	IP	↑ Insulin signaling pathway in adipocytes and liver	Dialyzed aqueous extract	Vijayakumar et al. (2005)
Alloxan‐induced diabetes in rat model	Seeds	5%	Oral	↑ Insulin release from residual β cells	Seed powder	Mohammad, Taha, Bamezai, and Baquer (2006)
Alloxan‐induced diabetes in rat model	Seeds	5 g	Oral	Restoration of membrane fluidity, Ca^2+^ levels; reduction of lipid peroxidation	Powder	Kumar, Kale, McLean, and Baquer (2012)
Alloxan‐induced diabetes in rat model	Seeds	9 g/kg	Oral	Undefined	Aqueous extract	Tripathi and Chandra (2010)
Alloxan‐induced diabetes in dogs	Seeds	1.5–2 g/kg	Oral	High percentage of dietary fibers in defatted extract	Lipid; defatted extract	Sauvaire et al. (1984)
Alloxan‐induced diabetes in dogs	Seeds	1.5–2 g/kg	Oral	Presence of fibers in defatted extract	Lipid; defatted extract	Valette et al. (1984)
Alloxan‐induced diabetes in rat model	Leaves	Aqueous (IP‐0.06–1; oral‐1–8 g/kg); ethanolic (IP‐0.8 g/kg)	IP and oral	Undefined	Aqueous; ethanolic extract	Abdel‐Barry et al. (1997)
Alloxan‐induced diabetes in mice model	Seeds	40 mg/kg	Oral	Regeneration of pancreatic β cells; adipogenesis	Aqueous alcohol extract	Shah et al. (2009)
Alloxan‐induced diabetes in rat model	Seeds	5%	Oral	Partial restoration of PK and PEPCK expression in liver; normalization of GLUT4 distribution in muscle	Powder	Mohammad, Taha, Akhtar, et al. (2006)
Alloxan‐induced diabetes in rat model	Seeds	5%	Oral	↓ G6Pase and FBPase either by c'AMP or ↓ glycolysis and gluconeogenesis	Powder	Gupta et al. (1999)
Alloxan‐induced diabetes in mice model	Seeds	Decoction (0.5 mL of 40%–80% decoction); ethanol (200–400 mg/kg)	Oral	Undefined	Decoction; ethanol extract	Ajabnoor and Tilmisany (1988)
Alloxan‐induced diabetic rat brain model	Seeds	5%	Oral	↑ GLUT4 expression in brain; restoration of MAO activity and DNA degradation	Powder	Kumar, Kale, and Baquer (2012)
Alloxan‐induced diabetes in rat model	Seeds	2–8 g/kg	Oral	Presence of high dietary fiber content	Powder	Khosla et al. (1995)
Alloxan‐induced diabetes in rat model	Seeds	1–4 g/kg	Oral	Undefined	Alcoholic extract	Vats et al. (2002)
Alloxan‐induced diabetes in rat model	Seeds	2 g/kg	Oral	↑ Glycemic status, SOD; ↓TBARS	Crude powder extract	Vanitha et al. (2012)
Alloxan‐induced diabetics in rat model	Seeds	100 mg/kg	Oral	Undefined	Methanolic extract	Dholi et al. (2011)
Alloxan‐induced diabetics in rat model	Seeds	50 mg/kg	Oral	Presence of alkaloids	Alkaloid extract	Patil et al. (2009)
Alloxan‐induced diabetes in rabbit model	Seeds	7.7 mL/12 h	Oral	Undefined	Ethanolic extract	Al‐Khateeb et al. (2012)
Alloxan‐induced diabetes in rabbit model	Seeds	200 mg/kg	Oral	Undefined	Ethanolic extract	Jawad and Hassan (2015)
Alloxan‐induced diabetes in rat model	Seeds	5%	Oral	↓ Blood glucose; restoration of G6PD, ME, ICDH, lipogenic enzymes (ATP‐citrate lyase and fatty acid synthase) activity	Powder	Yadav et al. (2004)
Alloxan‐induced diabetes in rabbit model	Seeds	300 mg/kg	Oral	Undefined	Aqueous extract	Kashif et al. (2023)
HFD‐induced insulin resistance in C57BL/6 mice model	Seeds	30–100 mg/kg	Oral	Modulation of GLUT‐2, GLUT‐4, IRS‐2 and SREBP‐1c expression	Oliosugar‐based standardized fenugreek seed extract	Kandhare et al. (2015)
HFD and alloxan‐induced diabetes in rat model	Seeds	42.33 mg/kg	Oral	↑ PDK1 and GLUT4 expression	Ethanol extract	Kan et al. (2017)
HFD‐ and STZ‐induced diabetes in C57BL/6J mice model	Seeds	20–80 mg/kg	Oral	↓ MDA; ↑ SOD, CAT activity	Petroleum ether; ethyl acetate; *n*‐butanol extracts	Li, Lu, et al. (2018)
High‐sucrose diet‐induced diabetes in rat model	Seeds	0.5 g/kg	Oral	↑ Insulin sensitivity by reduction of lipids levels	Powder	Muraki et al. (2011)
High‐energy diet and alloxan‐induced diabetes in rat model	Seeds	75, 35, and 15 mg/kg	Oral	Upregulation of IRS1 phosphorylation	Ethanol extract	Liu et al. (2016)
HFD‐induced diabetes in C57BL/6J mice model	Seeds	2 g/kg	Oral	Undefined	Hydroalcoholic extract	Hamza et al. (2012)
HFD diet‐induced diabetic in rat model	Seeds	50 mg/mL	Oral	↓ DPP‐IV; ↑ GLP‐1	Hot water extract	Ansari et al. (2021)
High‐fructose diet‐induced diabetes in rat model	Seeds	200 mg/kg	Oral	↑ Tyrosine phosphorylation of IRS, glucose uptake	Polyphenolic extract	Kannappan and Anuradha (2009)
HFD‐induced obese mice model, lipid loading test	Seeds	0.3–1%; 350–1500 mg/kg	Oral	↓ Lipid absorption	Ethanol extract	Handa et al. (2005)
Normal mice model	Seeds	50–400 mg/kg	Oral	Undefined	Methanolic extract	Syeda et al. (2014)
Normal mice model	Seeds	0.5–1.0 g/kg	Oral	Undefined	Aqueous and methanolic extract	Zia et al. (2001)

(C) In vitro and in vivo antidiabetic studies of isolated compounds of fenugreek							
Isolated compounds	Test model	Parts	Dose/concentration	Route of administration	Mechanism of action	Formulation	Ref.
Galactomannan	STZ‐induced diabetes in rat model	Seeds	500 mg/kg	Oral	Protection of β cells; ↑ insulin release, gut microbe balance; ↓ insulin resistance, oxidative stress	Ethanol extract	Jiang et al. (2017)
	Glucose‐induced diabetes in normal and diabetic rat model	Seeds	250 mg; only SDF extract, 10 mg	Oral	↓ Gastric emptying rate; delaying glucose absorption from the small intestine	Powder; water extract; Methanol extract; water extract of the methanol extractive‐free residue; SDF extract	Ali et al. (1995)
	STZ‐induced diabetes in rat model	Seeds	8%	Oral	↓ Maltase, lactase, sucrase, and lipase activity	Ethanol extract	Hamden, Jaouadi, et al. (2010)
	Alloxan‐induced diabetes in mice model	Seeds	50–200 mg/kg	Oral	Acting on the key enzymes of carbohydrate and lipid metabolism	Water‐soluble fractions	Kamble et al. (2013)
	Segments of jejunum and ileum from lean and obese rats	Seeds	0.1–0.5% (wt/wt)	In vitro	↓ Glucose uptake in the small intestine	Ethanol extract	Srichamroen et al. (2009)
GII	Alloxan‐induced diabetes in rabbit model	Seeds	25–175 mg/kg	Oral	Undefined	Water extract	Moorthy et al. (2010b)
	Alloxan‐induced diabetes in rabbit model	Seeds	50 mg/kg	Oral	↑ HK, GK, PK, ME, G6PD, SOD, and GPx; ↓ G6Pase, SDH, AR levels	Water extract	Puri et al. (2011)
	Alloxan‐induced diabetes in rabbit model	Seeds	50 mg/kg	Oral	Undefined	Water extract	Puri et al. (2012)
	Alloxan‐induced diabetes in rabbit model	Seeds	100 mg/kg	Oral	↑ PFK, PK, ↓ G6Pase, FBPase in the liver	Water extract	Moorthy et al. (2010a)
	Alloxan‐induced subdiabetic, mid‐diabetic rabbits, and severely diabetic rabbits	Seeds	50 mg//kg	Oral	↑ Serum insulin, sensitivity of tissue to insulin	Water extract	Puri et al. (2011)
4‐HIL	Fructose‐fed rats and STZ‐induced diabetes in rat model	Seeds	50 mg/kg	Oral	Undefined	Undefined	Haeri et al. (2009)
	Euglycemic hyperinsulinemic clamp studies; PI3‐kinase activity; Chronic treatment of extract on rats	Seeds	18–100 mg/kg	IV; IP	↑ Insulin response to glucose in β cells, activation of PI3K activity	Undefined	Broca et al. (2004)
	Rat pancreas; rat islets and human pancreas	Seeds	100–1000 μM	Cell line	↑ Insulin secretion through a direct action on β cells	Ethanol/water extraction	Sauvaire et al., (1998)
	High‐sucrose diet‐fed and STZ‐induced diabetes in rat model and L6 myotubes	Seeds	10 μM; 50 mg/kg	Cell line; Undefined	↑ AMPK and Akt; upregulation of the PGC‐1α, PGC‐1β, CPT, 1 and CPT 2 genes	Alcohol extract	Rawat et al. (2014)
	TNF‐α‐induced insulin resistance model using HepG2 cells	Seeds	5–20 μM	Cell line	↓ JNK, IRS‐1 (Ser^307^) phosphorylation, GSK‐3 phosphorylation; ↑AKT (Ser^473^)	Purchased	Lu et al. (2015)
	IVGTT (Nicotinamide and STZ‐induced diabetes in rat model); OGTT in dogs and rat model; In vitro (rat islets)	Seeds	IVGTT‐50 mg/kg; OGTT‐ dogs – 18, rats – 18‐36 mg/kg; In vitro (200 μM)	IV; Oral; Cell line	↑ β‐cell function	Linear and lactonic form (Purchased)	Broca et al. (1999)
	C57BL/KsJ‐db/db mice	Seeds	50 mg/kg	Oral	↑ Insulin sensitivity, glucose uptake in peripheral tissue	Aqueous extract	Singh et al. (2010)
	Fatty acid (palmitate)‐induced insulin resistance in L6 myotubes	Seeds	25 μM	Cell line	Restoration of IRS1/AKT/AS160/GSK pathway; ↑ GSK‐3β phosphorylation at Ser‐9; ↓ NF‐κB, JNK1/2, ERK1/2, p38 MAPK, ROS	Undefined	Maurya et al. (2014)
	HFD‐induced diabetic obese mice model; lipid loading test	Seeds	0.3%–1%; 350–1500 mg/kg	Oral	↓ Lipid absorption	Ethanol extract	Handa et al. (2005)
	Alloxan‐induced diabetes in mice model; pancreatic ductal stem cell culture	Seeds	40 mg/kg; 100–800 μL of 5 mM	Oral; cell line	Regeneration of pancreatic β cells; adipogenesis	Aqueous alcohol extract	Shah et al. (2009)
	STZ‐induced diabetic rats; Nonenzymatic glycosylation of hemoglobin assay	Seeds	200–400 mg/kg; 1 mL	Oral; in vitro	Undefined	Methanol extract	Devi and Raju (2020)
	HFD‐induced diabetes in Golden Syrian hamsters	Seeds	50 mg/kg	Oral	↓ TG, TC; ↑ HDLc/TC ratio	Alcoholic extract	Narender et al. (2006)
	STZ‐induced diabetic rats; Healthy rats; HEK‐293 cell line	Seeds	100 mg/kg; 1, 10, and 25 μm	Oral; cell line	↑ Insulin expression	Undefined	Singh et al. (2022)
Quercetin	High‐fructose diet‐induced diabetes in rat model	Undefined	50 mg/kg in 0.05% dimethyl sulfoxide	Oral	↑ Tyrosine phosphorylation of IRS, glucose uptake	Purchased	Kannappan and Anuradha (2009)
Diosgenin	STZ‐induced diabetes in rat model	Undefined	15–60 mg/kg	Oral	↑ Insulin production from residual β cells, HK; ↓ G6Pase, FBPase	Purchased	Saravanan et al. (2014)
	HFD‐ and STZ‐induced diabetes in rat model	Undefined	60 mg/kg	Oral	Improvement of metabolic dysregulation of lipid profile	Purchased	Naidu et al. (2015)
	Rabbit intestinal brush border membrane vesicle; Isolated hepatocyte suspension	Seeds	3.3–1.65 mg/mL	In vitro	↓ SGLT‐1‐mediated glucose absorption; ↓ glucagon‐induced HGPa activity	Undefined	Al‐Habori et al. (2001)
Trigonelline	Isolated hepatocyte suspension	Seeds	3.3–1.65 mg/mL	In vitro	↓ SGLT‐1‐mediated glucose absorption; ↓ glucagon‐induced HGPa activity	Undefined	Al‐Habori et al. (2001)
	Alloxan‐induced diabetes in rabbit model	Seeds	10 mg/12 h	Oral	Undefined	Ethanolic extract	Al‐Khateeb et al. (2012)
	Alloxan‐induced diabetes in rat model	Seeds	50 mg/kg	Oral	Protection of β cells; ↓ α amylase, maltase, lipase activity	Undefined	Hamden et al. (2013)
N55 ((9Z,12Z)‐N‐((3R,4R,5S)‐4,5‐dimethyl‐2‐oxotetrahydrofuran‐3‐yl) octadeca‐9,12‐dienamide)	In vitro	Seeds	Different concentrations	In vitro	Binds with GLP‐1 amide; ↑ potency in stimulating the cAMP pathway	Ethanol extract	King et al. (2015)
	C57BL/6 mice; In vitro	Seeds	0.6–5.4 μmol/kg	IP; in vitro	↑ Physiological level of GLP‐1 according to physiological need; less disruption of GLP‐1R signaling	Ethanol extract	Chou et al. (2017)
Rhaponticin	3T3‐L1 adipocytes	Seeds	Different concentrations	Cell line	↑ Activation of Akt and AMPK	*n*‐butanol extracts	Li, Luan, et al. (2018)
Orientin; Vitexin; Isovitexin	HFD‐ and STZ‐induced diabetes in C57BL/6J mice model	Seeds	20–80 mg/kg	Oral	↓ MDA; ↑ SOD, CAT activity	Ethyl acetate extract	Li, Lu, et al. (2018)
Rhaponticin; Deoxyrhapontin; Emodin	HFD‐ and STZ‐induced diabetes in C57BL/6J mice model	Seeds	20–80 mg/kg	Oral	↓ MDA activity; ↑ SOD, CAT activity	*n*‐butanol extracts	Li, Lu, et al. (2018)
Rhaponticin; Desoxyrhaponticin; Rhapontigenin	High‐sugar and HFD‐induced diabetes in adult and larvae zebrafish model	Seeds	2.5 to 100 mg/kg	IP	↓mTORc1, PPARG; ↑GAPDH	*n*‐butanol extract	Gao et al. (2024)
Isoorientin	3 T3‐L1 adipocytes	Seeds	10 μM	Cell line	↓ PPARγ, C/EBPα 30 and FAS expression; reactivation of AKT and AMPK; improvement of mitochondrial dysfunction	Undefined	Luan et al. (2018)
(11*Z*)‐11‐ eicosenoic acid 2, 3‐ bis[((9*Z*, 12*Z*, 15*Z*)‐1‐oxo‐9, 12, 15‐octadecatrien‐1‐yl)oxy] propyl ester	Alloxan‐induced diabetes in rat model	Seed oil	50 mg/kg	Oral	↑ Insulin sensitivity; ↓ α‐amylase, lipase activity; restoration of ACE activity	Hexane extract	Hamden et al. (2017)
Saponins	Alloxan‐induced diabetes in rat model	Seeds	100 mg/kg	Oral	Improve liver glycogen and body weight	Saponin fraction (ethyl alcohol extract)	Bansode, Salalkar, et al. (2017)
	Lysed diabetic human whole blood	Seeds	25–200 μg	In vitro	Undefined	Chloroform extract	Bansode, Gupta, et al. (2017)
22β‐acetoxyolean‐12‐ene‐ 3β, 24‐diol; Soyasapogenol B; Isonarthogenin	α‐glycosidase activity evaluation	Seed	20 μL	In vitro	↓ α‐ glycosidase activity	Acid hydrolyzed products of saponins (ethyl alcohol extract)	Zhang et al. (2020)
(25*R*)‐5‐en‐spirostane‐3β‐ol 3‐O‐β‐d glucopyranosyl‐(1 → 4)‐β‐d‐glucopyranoside; (25*R*)‐5‐en‐spirostane‐2α,3β‐diol 3‐O‐α‐L‐rhamnopyranosyl‐(1 → 2)‐[α‐L‐rhamnopyranosyl‐(1 → 4)]‐β‐d‐glucopyranoside; (25*R*)‐5‐en‐spirostane‐3β‐ol 3‐O‐β‐d glucopyranosyl‐(1 → 4)‐β‐d glucopyranoside	α‐glycosidase activity evaluation	Seed	20 μL	In vitro	↓ α‐glycosidase activity	Hydrolysis product (ethyl alcohol extract)	Zhang et al. (2020)

### 3.1 Preclinical (in vitro and in vivo) antidiabetic studies of fenugreek

Type 2 DM is primarily characterized by insulin resistance which is a state of defective insulin signaling (Burillo et al., 2021). Due to the abnormal insulin response, the glucose absorption from the bloodstream hampers, thereby increasing the blood glucose level (Yu et al., 2017). Impaired insulin response in liver, muscle, and adipose tissue plays a vital role in developing insulin resistance (Fazakerley et al., 2019). In target tissues, glucose is metabolized by two pathways: the phosphatidylinositol‐3‐kinase (PI3K)/Akt and the 5′‐AMP‐activated kinase (AMPK) pathways (Sharma et al., 2015; Yu et al., 2017). In the PI3K/Akt pathway, proteins including PI3K, protein kinase B (PKB/Akt), glucose transporter type‐4 (GLUT4), glucose transporter type‐2 (GLUT2), and glycogen synthase (GS) play vital roles for glucose homeostasis (Chadt & Al‐Hasani, 2020; Yu et al., 2017). Insulin binds with the insulin receptor (IR) of the target cells and activates the intrinsic tyrosine kinase activity that phosphorylates the insulin receptor substrate (IRS) (Rowland et al., 2011). The IRS acts as a docking site for PI3K that subsequently converts phosphatidylinositol‐4,5‐diphosphate (PIP_2_) to phosphatidylinositol‐3,4,5‐triphosphate (PIP_3_) at the plasma membrane (Leto & Saltiel, 2012). PIP_3_ further regulates phosphoinositide‐dependent kinase 1 (PDK1) phosphorylation that activates Akt, also known as PKB (Lankatillake et al., 2019). However, the expression of Akt is also associated with pancreatic β‐cell size and function (Tuttle et al., 2001). In addition, the activated Akt initiates the translocation of GLUT‐4 vesicles to the cell membrane and inhibits glycogen synthase kinase 3β (GSK‐3β). The inhibition of GSK‐3β is also associated with promoting glycogen synthase (GS), which increases glycogen synthesis from glucose (Rowland et al., 2011). However, the abnormalities in insulin‐stimulated Akt phosphorylation, impaired translocation of GLUT4 protein, and defective GS activity are often manifested in type 2 DM with insulin resistance (Biensø et al., 2012). Another pathway is the AMPK pathway which plays a significant role in metabolism and transport of glucose, oxidation of fatty acids and mitochondrial biogenies in skeletal muscle, suppression of hepatic glucose output, and inhibition of the expression of gluconeogenic genes in the liver (Yamada et al., 2010; Zhang et al., 2009). The activity of AMPK is altered in both animals and humans with type 2 DM. Its activation is connected to the sensitivity of insulin and glucose homeostasis (Zhang et al., 2009).

Several reports have been found that investigate the relationship between the PI3K/Akt pathway and fenugreek. Vijayakumar et al. (2005) first reported that the dialyzed aqueous extract of fenugreek seeds increased tyrosine phosphorylation of IR, IRS1, the p85 subunit of PI3K, and translocation of GLUT4. However, the rise of GLUT4 translocation was associated with protein kinase C (PKC) and the uptake of glucose was related to dose (maximum uptake, 25 μg/mL). This study was performed on cell lines including CHO‐HIRc‐mycGLUT4eGFP, 3T3‐L1‐mycGLUT4, 3T3‐L1, HepG2, and A431. In addition, 15 mg/kg of fenugreek extract reduced the glucose level by 50% in alloxan‐induced diabetic mice (Vijayakumar et al., 2005). Liu et al. (2016) investigated a polyherbal formulation of mulberry leaf, fenugreek seeds, and *Cinnamon cassia* extracts (6:5:3) on alloxan and high‐fat diet (HFD) induced diabetic mice and found that the formulation improved the IRS1 phosphorylation similar to the normal group (Liu et al., 2016). According to Kan et al. (2017), fenugreek seed boosted glucose absorption and insulin sensitivity in human adipocyte cell lines at submaximal insulin levels at lower dosages of 3 and 11 g/mL. However, on HFD and alloxan‐induced diabetic rats, the tested polyherbal formulation containing fenugreek seeds, American ginseng, and mulberry leaf extracts (42.33, 169.33, and 84.66 mg/kg, respectively) significantly reduced the lowered PDK1 and GLUT4 expression in adipose tissue. In addition, GLUT4 translocation was increased in alloxan‐induced diabetic rats (Kan et al., 2017). Another study reported that the oral administration of 5% fenugreek seeds powder restored GLUT4 expression in alloxan‐induced diabetic rats' brain (Kumar, Kale, & Baquer, 2012). In an earlier study conducted by Kumar et al. (2005), it was observed that GLUT4 expression and membrane distribution were considerably elevated in alloxan‐induced diabetic rats given 5% fenugreek seed powder mixed with the diet (Kumar et al., 2005). Mohammad, Taha, Akhtar, et al. (2006) found that fenugreek seed powder increased the translocation of GLUT4 to the plasma membrane in alloxan‐induced diabetic rats.

**FIGURE 2:**
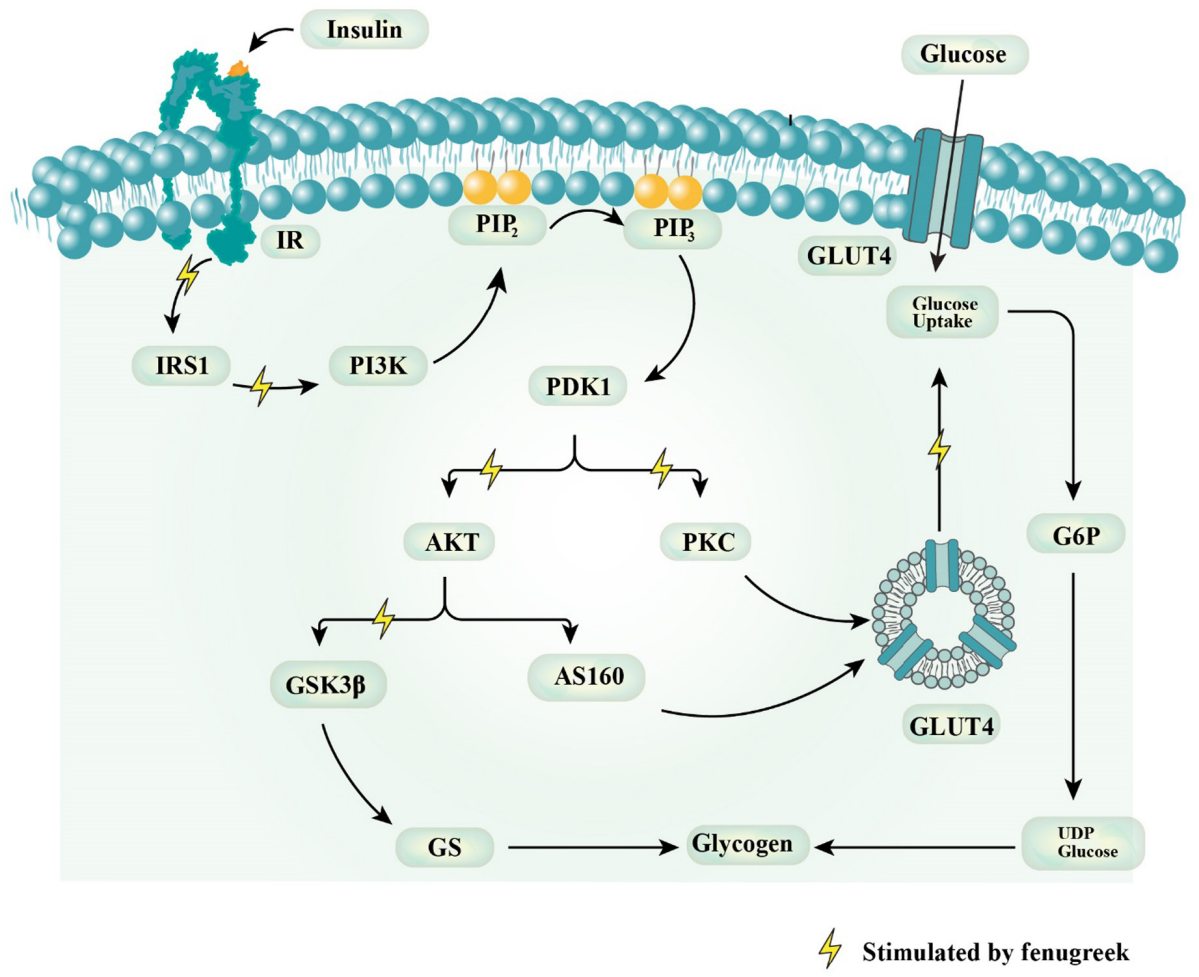
Proposed mechanism of action of fenugreek on PI3K/Akt pathway. Yellow color indicates the pathways stimulated by fenugreek and its isolated compounds.

Two studies reported the glucose uptake of fenugreek seeds and leaves in streptozocin (STZ)‐induced diabetes rats (Arshadi, Ali Azarbayjani, et al., 2015; Devi et al., 2003). Aqueous extracts of fenugreek seeds at 0.8 g/kg and 1.6 g/kg, along with swimming, significantly improved insulin resistance by modulating GLUT4 signaling pathways (Arshadi, Ali Azarbayjani, et al., 2015). Apart from seeds, fenugreek leaves at 0.5 and 1 g/kg reactivated the GS system, improving the glycogen content (Devi et al., 2003). Furthermore, oligosaccharide‐based fenugreek seed extract administration at 30, 60, and 100 mg/kg increased the expressions of GLUT2 and GLUT4 and decreased sterol regulatory element binding protein 1c (SREBP‐1c) expressions in the adipose tissue and liver of HFD‐induced insulin resistance in mice (Kandhare et al., 2015). A probable mechanism of fenugreek to control the pathway of PI3K/Akt is given in Figure 2.

Among the bioactive compounds, 4‐hydroxyisoleucine (4‐HIL) is mostly studied as an insulinotropic amino acid in fenugreek seeds. It was reported earlier that 4‐HIL increased insulin sensitivity and reduced insulin resistance by activating PI3K activity in both the liver and the muscle of Zucker fa/fa and sucrose‐lipid diet rats (Broca et al., 2004). Lu et al. (2015) reported that 4‐HIL at 20 μM on HepG2 cells inhibited tissue necrosis factor α (TNF‐α) induced JNK and IRS‐1 (Ser^307^) phosphorylation, whereas it increased AKT (Ser^473^) and GSK‐3 phosphorylation, resulting in improved hepatic insulin resistance (Lu et al., 2015). Maurya et al. (2014) found that 4‐HIL restored the altered phosphorylation of IRS‐1, AKT (Ser^473^), AS160 (Thr^642^), GSK‐3, and GLUT4 translocation, as well as inhibited reactive oxygen species (ROS) production and NF‐κB and MAPK activation in palmitate‐induced insulin resistance in L6 skeletal muscle cells (Maurya et al., 2014). 4‐HIL was also reported to increase AMPK, pAMPK, GLUT4, AKT, and pAKT levels in both high sucrose diet‐fed STZ‐induced diabetic rats and L6 myotubes by Rawat et al. (2014). In addition, in the L6 myotubes, the uptake of glucose increased by 87.3%. Furthermore, 4‐HIL also upregulated gene expression related to mitochondrial biogenesis and energy metabolism (PGC‐1α, PGC‐1β, CPT1, and CPT2) (Rawat et al., 2014). Quercetin, a flavonoid isolated from fenugreek seeds, and polyphenolic extract of seeds at 50 mg/kg in 0.05% dimethyl sulfoxide and 200 mg/kg, respectively, activated protein tyrosine kinase (PTK) activity and reduced protein tyrosine phosphatase (PTP) activity result in enhanced tyrosine phosphorylation in high‐fructose diet‐induced insulin resistance in rats (Kannappan & Anuradha, 2009). Another flavonoid of fenugreek seeds, isoorientin, increased glucose uptake by increasing Akt and AMPK phosphorylation, which resulted in improved mitochondrial function in the insulin resistance model of 3 T3‐L1 adipocyte cells. Furthermore, isoorientin and vitexin also reduced adipocyte differentiation and accumulation of lipid by inhibiting the transcription of adipocyte‐specific genes, including PPARγ, AP2, C/EBPα, SREBP1c, and FAS. In this study, four flavonoid glycosides were isolated from fenugreek seeds, including orientin, isoorientin, vitexin, and isovitexin, and used at a concentration of 10 μM (Luan et al., 2018). Polyphenol stilbenes (rhaponticin, desoxyrhaponticin, and rhapontigenin), isolated from the *n*‐butanol extracts of fenugreek seeds, also enhanced insulin sensitivity by increasing PKB/Akt and AMPK phosphorylation, as well as increased mitochondrial function in the insulin resistance model of 3 T3‐L1 adipocyte cells. However, rhaponticin was the most active of the three compounds (Li, Luan, et al., 2018). In a recent study conducted by Gao et al. (2024), stilbenoids (rhaponticin, desoxyrhaponticin, and rhapontigenin), isolated from fenugreek seeds, improved insulin resistance through modulating the PI3K/Akt/mTOR signaling pathway. These compounds at 2.5 to 100 mg/kg reduced the expression of mTORc1, and PPARγ and increased glyceraldehyde 3‐phosphate dehydrogenase (GAPDH) level compared to the control, where rhaponticin was reported as the most promising compound, in the high‐sugar and HFD‐induced diabetes in adult and larvae zebrafish model (Gao et al., 2024).

Alterations in glucose utilizing and metabolizing enzyme activities are common manifestations of insulin resistance (Noguchi et al., 2013; Wu et al., 2005). Insulin generally controls glucose homeostasis by promoting glycogen synthesis in muscle and liver, decreasing hepatic glucose production and output (inhibition of gluconeogenesis and glycogenolysis), and increasing glucose uptake, particularly in adipocytes and muscle (Leszek, 2017; Li et al., 2022). Several studies have reported that fenugreek restored the altered glucose‐utilizing and metabolizing enzyme activities. Vats et al. (2003) reported that 2 mg/kg of defatted extract of fenugreek seeds significantly improved the hepatic phosphofructokinase (PFK), glucokinase (GK), and skeletal hexokinase (HK) activities compared to the control but could not normalize their values to the euglycemic condition (Vats et al., 2003). In alloxan‐induced diabetic mice, the GK and HK activities were also reported to increase in the liver by 4.6 and 1.5 fold, respectively, after administration of 15 mg/kg of dialyzed aqueous extract (Vijayakumar & Bhat, 2008). Gad et al. (2006) found that the aqueous extract of fenugreek seeds at 1.5 g/kg suppressed glucose‐6‐phosphatase (G6Pase) activity by 32% and enhanced glucose‐6‐phosphate dehydrogenase (G6PD) activity by 31% in STZ‐induced diabetic rats. These researchers noted that that this extract failed to change the PFK enzyme activity in the liver compared to control (Gad et al., 2006). On the other hand, another study reported that the water extract of fenugreek seeds at 2 mg/mL increased 6‐phosphofructo‐1‐kinase (6PFK1) activity significantly in both the liver and intestinal mucosa by 54% and 75%, respectively, in STZ‐induced diabetic rats (Ali et al., 2013). Apart from these, Bera et al. (2013) described that a combination of aqueous extracts of *Psoralea corylifolia* seeds and *Trigonella foenum‐graecum* seeds (1: 1) at 200 mg/5 mL restored the HK, G6PD, and G6Pase activity to normal levels in the liver of STZ‐induced diabetic rats (Bera et al., 2013). An earlier study conducted by Gupta et al. (1999) reported that fenugreek seed powder at 5% nearly restored G6Pase and fructose‐1,6‐bisphosphatase (FBPase) activities in the liver and kidney of alloxan‐induced diabetic rats. However, optimum activity was mentioned after the use of a combination of vanadate and fenugreek seed powder (Gupta et al., 1999). Five‐percent fenugreek seed powder was also noted to partially restore the altered pyruvate kinase (PK) and phosphoenolpyruvate carboxykinase (PEPCK) enzyme activities in the liver in alloxan‐induced rats. Additionally, this powder also restored the GLUT4 distribution in skeletal muscle (Mohammad, Taha, Akhtar, et al., 2006). Aside from the seeds, we found one earlier study on fenugreek leaves. Devi et al. (2003) described that fenugreek leaves powder at 0.5 and 1 g/kg increased HK activity and suppressed both G6Pase and FBPase enzyme activities in the liver and kidney of STZ‐induced diabetic rats, and the effect was similar to glibenclamide (Devi et al., 2003).

Four studies were found that evaluated the antidiabetic effect of isolated compounds from fenugreek, focusing on glucose utilizing and metabolizing enzymes. However, among them, two studies reported the effect of a compound GII, purified from the water extract of fenugreek seeds, whose structure is not yet solved, on alloxan‐induced subdiabetic, moderately diabetic, and severely diabetic rabbits (Moorthy et al., 2010a; Puri et al., 2011). Moorthy et al. (2010a) investigated GK, PFK, PK, G6Pase, and fructose 1,6‐diphosphatase/ FBPase (FDPase) enzyme activities at a dose of 100 mg/kg GII on subdiabetic and moderately diabetic rabbits. The glycolysis enzyme activities were evaluated in the liver and muscle, whereas the gluconeogenic enzyme activities were assessed in the liver and kidney. Except from the liver, in the muscles of both subdiabetic and moderately diabetic rabbits, GK activity was increased by 35 and 40%, respectively. In addition, both the liver and muscle of subdiabetic rabbits demonstrated an improved PFK and PK (13–50%) level. However, these two enzymes were moderately elevated (18–23%) in moderately diabetic rabbits. Furthermore, the G6Pase and FDPase activity mostly decreased by 22, and 32%, respectively, in the liver of moderately diabetic rabbits. On the other hand, in subdiabetic rabbits, both enzymes were only reduced in the liver (15%–20%) (Moorthy et al., 2010a). Puri et al. (2011) also investigated the effect of GII at a dose of 50 mg/kg on subdiabetic, moderately diabetic, and severely diabetic rabbits. GII restored the altered level of HK in the livers of subdiabetic and moderately diabetic rabbits but failed to normalize the HK level in the muscles of moderately diabetic rabbits and in the liver and the muscles of severely diabetic rabbits. GK activity increased closer to the normal range in the livers of subdiabetic, moderately diabetic, and severely diabetic rabbits (Puri et al., 2011). PK activity in this study did not change significantly in the liver, kidney, and muscle compared to control in subdiabetic untreated and treated rabbits, which was also similar to the previous study that reported a moderate increase of PK (13%) in the liver of subdiabetic rabbits (Moorthy et al., 2010a; Puri et al., 2011). Quercetin and polyphenolic extract of fenugreek seeds at a dose of 50 mg/kg in 0.05% dimethyl sulfoxide and 200 mg/kg, respectively, also increased HK and PK activities while suppressing G6Pase and FBPase activities in high‐fructose‐fed rats (Kannappan & Anuradha, 2009). Diosgenin at 30 mg/kg also significantly increased HK activity in the serum, muscle, and kidney of STZ‐induced diabetic rats. In addition, the G6Pase activity in serum and kidney and the FBPase activity in serum, muscle, and kidney were reduced to normal (Saravanan et al., 2014).

Pancreatic β‐cell function also plays a key role in the development of diabetes. In the prediabetic state, β‐cells manage insulin resistance by increasing insulin release (Khin et al., 2023). However, in chronic conditions, apoptosis of the β‐cell increases; thereby, mass and function of the β‐cell reduce gradually, deteriorating insulin secretion (Khin et al., 2023; Remedi & Emfinger, 2016). Fenugreek seed oil at 10% in food was reported to prevent β‐cell damage, decrease proinflammatory cytokine interleukin‐6 and glucose level (by 48%), and increase plasma insulin level by 97% compared with control in alloxan‐induced diabetic rats (Hamden, Masmoudi, et al., 2010). Another study also reported that fenugreek essential oil 5% in combination with omega‐3 fatty acids restored the β‐cell structure and increased insulin secretion in alloxan‐induced diabetic rats (Hamden et al., 2011). Furthermore, Mohamed et al. (2015) described that the aqueous extract of fenugreek seeds at 100 mg/kg increased the β‐cell numbers and reduced β‐cell diameter in alloxan‐induced obese diabetic rats. However, this study also suggested that a combination of fenugreek, nigella, and termis seeds was more effective for regeneration of the β cells and improvement of insulin levels compared to fenugreek alone (Mohamed et al., 2015). Another recent study also reported that fenugreek seeds, 10% alone or in combination with onion, also simulated β‐cell regeneration, restored the normal level of β‐cell mass, increased insulin secretion and sensitivity, and partially restored insulin resistance in STZ‐induced diabetic rats (Pradeep & Srinivasan, 2017). Fenugreek seeds also improve the condition of residual β cells. An earlier study conducted on alloxan‐induced diabetic rats found that the seed powder at 5% w/w stimulated the residual β cells to secrete insulin and decreased the blood glucose level to 125 ± 9.8 mg/dL (Mohammad, Taha, Bamezai, & Baquer, 2006).

Regarding isolated compounds, galactomannan, derived from the ethanol extract of fenugreek seeds, at 0.5 g/kg reduced insulin resistance by regulating the tryptophan metabolism, protecting islet cells, and improving insulin secretion through controlling the arachidonic acid metabolism in STZ‐induced diabetic rats (Jiang et al., 2017). Diosgenin at 60 mg/kg also showed antidiabetic activity through the stimulation of insulin secretion from the existing β cells in STZ‐induced diabetic rats (Saravanan et al., 2014). Several studies have found that 4HIL increases insulin secretion only in the presence of high blood glucose. Sauvaire et al. (1998) first reported that 4‐HIL, at a concentration of 100 μmol/L to 1 mmol/L, stimulated pancreatic β cells to release more insulin secretion, only in the presence of supranormal levels (6.6–16.7 mmol/L) of glucose in isolated perfused rat pancreas, rat islets, and human islets. This study also mentioned that 4HIL had no effect on pancreatic glucagon, somatostatin, or other insulinotropic substances, including leucine, arginine, tolbutamide, and glyceraldehyde (Sauvaire et al., 1998). Another study conducted by Broca et al. (1999) also reported that 4HIL was ineffective at low glucose concentration (3 mM) and increased insulin release only at high glucose concentration (16.7 mM) in rat islets. In addition, in both in vitro and in vivo studies (18, 36, and 50 mg/kg in dogs), 4HIL stimulated pancreatic β‐cell function directly, suppressed basal hyperglycemia, and improved glucose tolerance. However, this study also mentioned that only the linear form (from plant extraction) of 4HIL showed insulinotropic activity (Broca et al., 1999). Furthermore, Shah et al. (2009) found that 4HIL demonstrated islet neogenesis from pancreatic duct cells and adipogenesis in a concentration‐dependent manner in alloxan‐induced diabetic rats. The insulin levels in both the pancreas and serum increased after treatment with 4HIL (Shah et al., 2009). A recent study also reported that 4HIL increased insulin expression at a dose of 100 mg/kg in STZ‐induced diabetic rats (Singh et al., 2022). Apart from these, flavonoids of the ethanol extract of fenugreek seeds at 0.05 g/1 mL of water restored altered lipid metabolism, decreased β‐cell damage, and increased serum insulin levels by 5.15 ± 0.88 mU/L compared with healthy groups in STZ‐induced diabetic rats (Jiang et al., 2018).

The inhibition of α amylase and α glucosidase enzymes is another common therapeutic approach for reducing postprandial hyperglycemia (Kalita et al., 2018). α‐amylase, which is present in both saliva and pancreatic juice, catalyzes the hydrolysis of the α‐1,4 glucan linkages of starch, maltodextrins, and other carbohydrates (Gong et al., 2020). On the other hand, α glucosidase is present in the brush border of human intestinal mucosal cells and promotes the hydrolysis of α‐1,4 glycosidic linkages of carbohydrates (Khosravi et al., 2020; Teng & Chen, 2017). Generally, polysaccharides are converted into oligosaccharides or disaccharides by α amylase, which are further converted into monosaccharides by α glucosidase (Teng & Chen, 2017). Gad et al. (2006) reported that the aqueous extract of fenugreek seeds inhibited α amylase activity with an IC_50_ value of 1.3 mg/mL. In the STZ‐induced diabetic rats, they found that the fenugreek extract at 1.5 g/kg suppressed the digestion and absorption of glucose (Gad et al., 2006). The terpene‐rich fraction, extracted from fenugreek oil, in alloxan‐induced diabetic rats at a dose of 5%, along with the omega‐3 fatty acids, was also reported to reduce the α‐amylase and the maltase enzyme activity in pancreas by 46 and 37%, respectively, and in plasma by 52 and 35%, respectively. Three compounds were present in the highest amount: neryl acetate (17%), camphor (16%), and β‐pinene (15%). However, this study highlighted that β‐pinene was mostly responsible for the inhibition of both enzyme activities (Hamden et al., 2011). Kan et al. (2017) found that the ethanolic extract of fenugreek seeds had slight α glucosidase inhibition activity. The reported extract inhibited α amylase activity with an IC_50_ of 73.2 μg/mL, whereas it failed to suppress α glucosidase enzyme activity (Kan et al., 2017). Arooj et al. (2024) reported that the boiled and unboiled fenugreek seed extract at 9% (w/v) reduced the α‐amylase activity by 61.6% and 58.4%, respectively. Similarly, the unboiled and boiled extracts inhibited α‐glucoside enzyme activity; the unboiled extract showed the highest inhibition (33.1%) (Arooj et al., 2024). In an earlier study conducted by Kumar et al. (2005) mentioned that fenugreek seed mucilage in combination with spent turmeric restored the levels of intestinal and renal maltase enzyme activities to normal (Kumar et al., 2005). Another earlier study also reported that fenugreek seeds reduced hyperglycemia in STZ‐induced diabetic rats, which was associated with inhibition of carbohydrate hydrolyzing enzymes in the intestine. In addition, fenugreek seeds also reduced the severity of the onset of diabetes in normal rats (Riyad et al., 1988).

We found several studies on the soluble dietary fiber (SDF) fraction and carbohydrate metabolizing enzyme activities of fenugreek. Hannan et al. (2007) reported that the SDF fraction of fenugreek seeds at 0.5 g/kg improved glucose homeostasis by reducing carbohydrate digestion and absorption in STZ‐induced diabetic rats (Hannan et al., 2007). The SDF fraction at 0.43% (w/v) also delayed amylolysis, and at ≥1% (w/v), it slightly retarded maltose transport in simulated intestinal digesta (Repin et al., 2017). Galactomannan is the main constituent of the SDF fraction which was also reported to prevent α‐amylase and α‐glucosidase enzyme activity with an IC_50_ value of 21.08 ± 0.085 and 67.17 ± 5.15 μg mL^−1^, respectively (Srinivasa & Naidu, 2021). In an earlier study, galactomannan was isolated from the ethanolic extract of fenugreek seeds and it decreased the maltase, sucrase, and lactase enzyme activities in STZ‐induced diabetic rats at 8% in food (Hamden, Jaouadi, et al., 2010). Furthermore, galactomannan was also reported to reduce weight gain and protect the pancreas by acting on key enzymes of carbohydrate and lipid metabolism in the liver in alloxan‐induced diabetic mice (Kamble et al., 2013).

A pure triglyceride isolated from hexane extract of fenugreek seeds, (11*Z*)‐11‐eicosenoic acid 2, 3‐ bis [((9*Z*, 12*Z*, 15*Z*)‐1‐oxo‐9, 12, 15‐octadecatrien‐1‐yl) oxy] propyl ester, at 50 mg/kg suppressed pancreatic α amylase activity by 36% in alloxan‐induced diabetic rats (Hamden et al., 2017). Trigonelline, an alkaloid present in seeds, at a dose of 50 mg/kg decreased maltase enzyme activities by 52% in the small intestine of alloxan‐induced diabetic rats (Hamden et al., 2013). The saponin and sapogenin fractions of fenugreek seeds demonstrated α glucosidase inhibitory activity (Zhang et al., 2020). Zhang et al. (2020) reported that sapogenin, which is a hydrolyzed product of saponin, manifested greater inhibition of α glucosidase compared to saponins due to the absence of sugar chains. Three sapogenins, including 22β‐acetoxyolean‐12‐ene‐3β,24‐diol, soyasapogenol B, and isonarthogenin, inhibited α glucosidase enzyme activity with an IC_50_ value of 15.18, 8.98, and 7.26 μM, respectively, compared to the IC_50_ of acarbose, 5.23 μM. On the other hand, (25*R*)‐5‐en‐spirostane‐3β‐ol 3‐O‐β‐d glucopyranosyl‐(1 → 4)‐β‐d‐glucopyranoside, and (25*R*)‐5‐en‐spirostane‐2α,3β‐diol 3‐O‐α‐l‐rhamnopyranosyl‐(1 → 2)‐[α‐l‐rhamnopyranosyl‐(1 → 4)]‐β‐d‐glucopyranoside were two saponins that inhibited α‐glucosidase enzyme activity with an IC_50_ value of 5.49 and 14.01 μM (Zhang et al., 2020).

Glucagon‐like peptide (GLP‐1)‐based therapy, including GLP‐1 receptor (GLP‐1R) agonists and dipeptidyl peptidase IV (DPP‐IV) enzyme antagonists, is also commonly used for diabetes (Lee & Jun, 2014). Following the intake of meals, two incretin hormones, glucose‐dependent insulinotropic polypeptide (GIP) and GLP‐1, are secreted from the intestine and control insulin secretion in a dose‐dependent manner (Ahrén, 2007; Seino et al., 2010). The GIP stimulates glucagon secretion rather than insulin; therefore, it is a less explored target for DM (Ahrén, 2007). On the other hand, GLP‐1 is considered a potential target for diabetes since it accelerates insulin biosynthesis, delays gastric emptying, inhibits glucagon secretion, and stimulates β‐cell neogenesis (Ahrén, 2007; Kshirsagar et al., 2011; Maselli & Camilleri, 2020). GLP‐1 activates the GLP‐1R, a G‐protein‐coupled receptor, which is present in a variety of tissues including the pancreas, heart, lungs, kidneys, neurons, blood vessels, and lymphocytes (Chou et al., 2017; Lee & Jun, 2014; Salvatore et al., 2007). After the intake of meals and nutrients such as carbohydrates, fats, protein, and dietary fiber, the endogenous form of GLP‐1 (GLP‐1 (7–36) amide) increases rapidly by threefold to fourfold (King et al., 2015; Lee & Jun, 2014). The bioactive form of GLP‐1 rapidly degraded to its inactive form, GLP‐1 (9–36) amide, due to the removal of the dipeptides by DPP‐IV enzyme (Arulmozhi & Portha, 2006; Lee & Jun, 2014). Although both GLP‐1R agonists and DPP‐IV inhibitors are considered potential approaches for diabetes, they are also associated with notable side effects, particularly GLP‐1R agonists. GLP‐1R agonists activate GLP‐1R globally and chronically, which causes side effects including gastrointestinal distress, nausea, and vomiting (Ahrén, 2007; King et al., 2015). Compound (9Z,12Z)‐N‐((3R,4R,5S)‐4,5‐dimethyl‐2‐oxotetrahydrofuran‐3‐yl) octadeca‐9,12 dienamide (N55), present in the seeds, modulated the GLP‐1R signaling positively without binding to the receptor, which could be a new approach for controlling diabetes (Chou et al., 2017; King et al., 2015). King et al. (2015) first reported that N55, isolated from the ethanolic extract of fenugreek seeds, selectively enhanced the potency of endogenous GLP‐1 (7–36) amide without activating the GLP‐1R in an in vitro study. The production of cAMP and GLP‐1R endocytosis was increased in a dose‐dependent manner, and N55 showed no effect on GIP and GLP‐1R agonists except exendin‐4 (King et al., 2015). Chou et al. (2017) also reported similar findings in an in vitro study, where N55 bound with GLP‐1 rather than GLP‐1R and activated GLP‐1 activity. In the C57BL/6 mice, this compound improved glucose tolerance according to the physiological requirements after intraperitoneal administration at a dose of 0.6, 1.8, and 5.4 μmol/kg (Chou et al., 2017).

**FIGURE 3:**
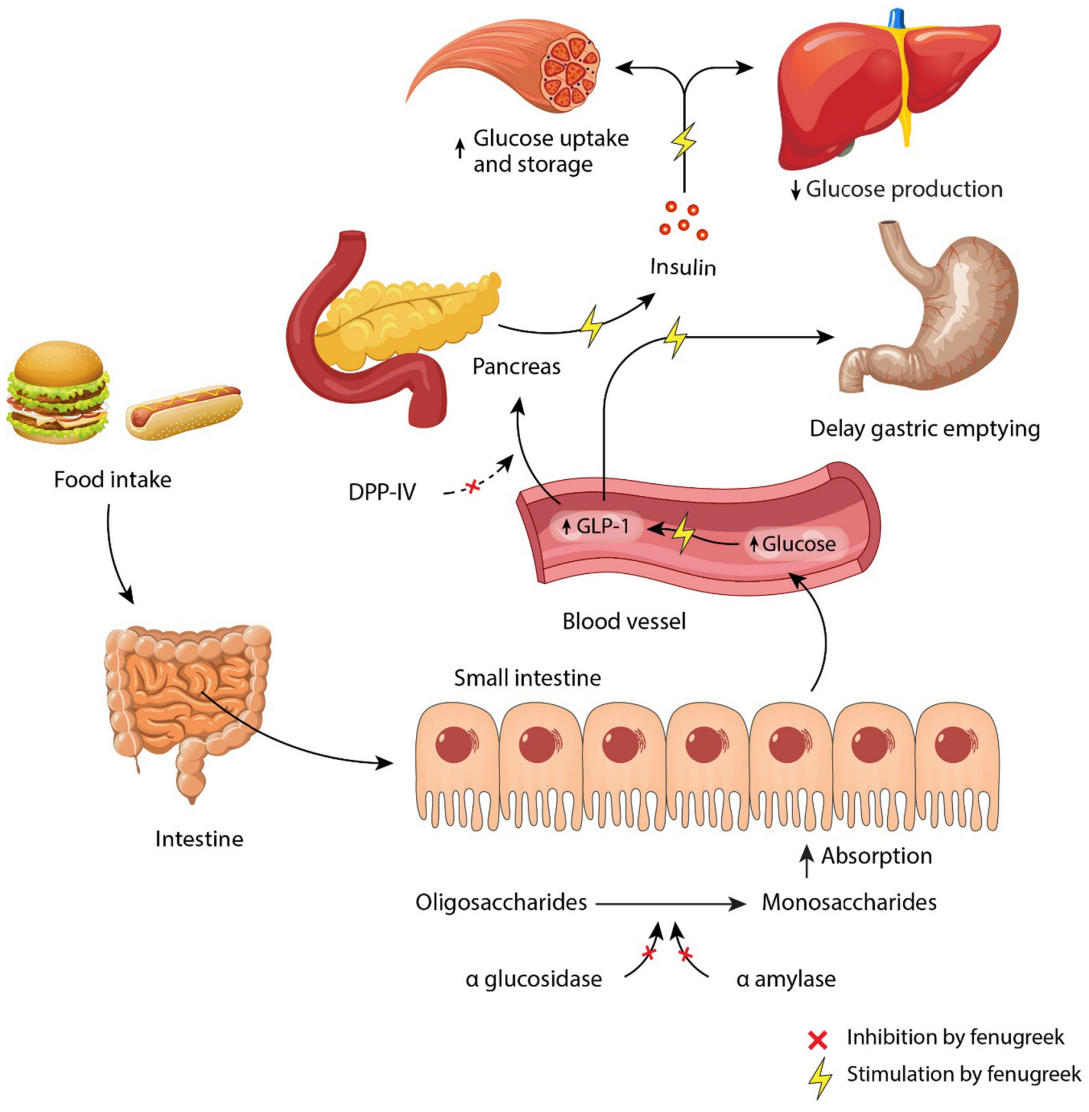
An overall proposed mechanism of action of fenugreek and its isolated compounds. Yellow color indicates stimulation and red color indicates the inhibition by fenugreek.

Regarding DPP‐IV enzyme inhibitors, one in vitro study was reported that the hot water extract of fenugreek seeds reduced the DPP‐IV enzyme activity by 28 ± 2% (IC_25_,4700 μg/mL) compared to sitagliptin, vildagliptin, and diproton A. However, in HFD‐induced diabetic rats at 250 mg/5 mL/kg, the bioactive form of GLP‐1 was increased to a lower extent than sitagliptin and vildagliptin (Ansari et al., 2021). In our recent *in silico* study, we found that isovitexin and deoxyrhapontin bound well with the DPP‐IV enzyme. In addition, isovitexin showed higher affinity for the DPP‐IV enzyme compared to sitagliptin (Sarker et al., 2023). However, a probable mechanism of action of fenugreek on glucose is given in Figure 3.

Diabetes is often associated with cardiovascular and renal complications such as coronary heart disease, stroke, and kidney failure (Harding et al., 2019). The angiotensin‐converting enzyme (ACE) inhibitor has been widely used as a first‐line therapy for cardiovascular and renal complications (Zhao & Schooling, 2021). However, few articles have been found on the ACE activity of fenugreek seeds. Hamden et al. (2011) reported that omega‐3 fatty acid with fenugreek terpenes significantly reduced plasma ACE activity in alloxan‐induced diabetic rats (Hamden et al., 2011). In another study, a triglyceride, (11*Z*)‐11‐eicosenoic acid 2, 3‐ bis [((9*Z*, 12*Z*, 15*Z*)‐1‐oxo‐9, 12, 15‐octadecatrien‐1‐yl) oxy] propyl ester, of fenugreek seed oil at 50 mg/kg reduced kidney and serum ACE activity by 33 and 22%, respectively, in alloxan‐induced diabetic rats. This study also described that such alterations were associated with interactions between fenugreek and disulfide bridges present on the ACE surface (Hamden et al., 2017).

The polyol pathway, which converts glucose to sorbitol, plays a significant role in the late onset of diabetic complications (Januzzi et al., 2023). Aldose reductase (AR) is the rate‐limiting enzyme of the polyol pathway, and its inhibition is reported to reduce diabetic complications (Januzzi et al., 2023). Here, we found one study reporting that oral administration of a purified compound of fenugreek seeds, GII, at 50 mg/kg reduced the levels of AR to normal in both the kidney and liver of alloxan‐induced subdiabetic, moderately diabetic, and severely diabetic rabbits (Puri et al., 2011).

Delay in gastric emptying is another mechanism that lies in the fact that slow exposure and slow absorption of glucose ultimately reduce the amount of insulin required for glucose disposal (Seifu et al., 2017). A previous study conducted by Ali et al. (1995) reported that galactomannan, a major constituent of the SDF fraction, significantly reduced postprandial hyperglycemia in glucose‐induced diabetic rats by delaying the rate of both gastric emptying and glucose absorption (Ali et al., 1995).

Diabetes is often associated with oxidative stress and the generation of advanced glycation end products (AGEs), which are a result of protein, nucleic acids, and lipid glycation (Mridula et al., 2021). Several studies reported that fenugreek exhibited antidiabetic and neuroprotective effects due to its antioxidant properties (Kumar, Kale, & Baquer, 2012; Li, Lu, et al., 2018). Li, Lu, et al. (2018) reported that the ethyl acetate and *n*‐butanol extracts of fenugreek seeds reduced malondialdehyde (MDA) activity and increased catalase (CAT) and superoxide dismutase (SOD) activity in both HFD‐ and STZ‐induced diabetic mice at doses of 20 and 80 mg/kg. Flavonoids (orientin, vitexin, and isovitexin) in ethyl acetate extract and stilbene glycosides (rhaponticin, deoxyrhapontin, and emodin) in *n*‐butanol extract were responsible for the effect (Li, Lu, et al., 2018). An earlier study also revealed that oral administration of fenugreek seed powder at 5% in food reduced both MDA activity and DNA degradation in alloxan‐induced diabetic rats (Kumar, Kale, & Baquer, 2012). The fine crude powder of fenugreek seeds at 2 g/kg was reported to reduce the blood glucose level due to the antioxidative activity of the seeds. The fenugreek seeds reduced the thiobarbituric acid reactive substances (TBARS) level and improved the SOD levels in alloxan‐induced diabetic rats (Vanitha et al., 2012). Fenugreek seed oil also demonstrated the improvement of cellular antioxidant defenses in alloxan‐induced diabetic rats (Hamden, Masmoudi, et al., 2010). Hamden et al. (2017) described that (11*Z*)‐11‐ eicosenoic acid 2, 3‐bis [((9*Z*, 12*Z*, 15*Z*)‐1‐oxo‐9, 12, 15‐octadecatrien‐1‐yl)oxy] propyl ester prevented the autooxidation of glucose and reduced AGEs formation, resulting in an increase in antioxidant activity (Hamden et al., 2017). Aside from these, Jiang et al. (2017) conducted a metabonomic study of galactomannan and reported that therapeutic pathways of galactomannan against hyperglycemia were associated with regulation of metabolism of histidine, tryptophan, phenylalanine, sphingolipid, glycerophospholipid, and arachidonic acid (Jiang et al., 2017).

### 3.2 Clinical studies of fenugreek and its isolated compounds

In this study, a total of 24 studies have been found on clinical trials of fenugreek. Most of these studies used fenugreek seeds, except for two studies, where one used the isolated compound galactomannan and another used leaves. Table 2 denotes the reported clinical study of fenugreek and its isolated compounds. Najdi et al. (2019) reported that fenugreek seeds at 2 g/day with metformin significantly suppressed fasting plasma glucose (FPG) and high‐density lipoprotein (HDL) to low‐density lipoprotein (LDL) ratio in comparison to the glibenclamide group. In addition, fasting blood glucose (FBG), glycated hemoglobin (HbA1c), triglyceride (TG), and LDL levels were also reduced but not as much as glibenclamide. This randomized trial was conducted on 12 uncontrolled DM patients for 3 months (Najdi et al., 2019). Ranade and Mudgalkar (2017) conducted a randomized study on 62 type 2 DM patients, where fenugreek seeds at 10 g/day in hot water along with previous antidiabetic medication, diet, control, and exercise were used. The fenugreek seed group significantly reduced FBG levels in the 5th month and decreased HbA1c levels in the 6th month, thereby showing a synergistic antidiabetic effect (Ranade and Mudgalkar, 2017). Kassaian et al. (2009) also reported that fenugreek seed powder in hot water significantly reduced FBG, TG, and very low‐density lipoprotein cholesterol (VLDL‐C) by 25%, 30%, and 30.6%, respectively. This study was conducted on 24 type 2 DM patients for 2 months, and 5‐g seed powder in one quart of hot water and 250 g of yogurt were used. This study noted that the yogurt fenugreek mixture had no significant effect (Kassaian et al., 2009).

**TABLE 2:** Clinical antidiabetic studies of fenugreek and its isolated compounds.

Study design	Number, gender (*n*), and characteristics of patients	Preparation of fenugreek/active molecules	Experimental intervention (dose, type, and duration)	Control intervention (dose, type, and duration)	Group design	Assessment	Ref.
15 patients at risk of type 2 DM	*n* = 15, M = 10; F = 5; mean age 55.1 years; FBG 5.5 mmol/L	Water extract (WE) of fenugreek gum	Maltose syrup‐ and starch‐based pudding, puddings contained fenugreek‐WE; concentration three times the apparent viscosity	Chocolate flavored puddings contained no SDF	Undefined	Investigated glycemic and insulinemic responses and gastric emptying.	Repin et al. (2017)
RCTs on 12 patients with uncontrolled type 2 DM	*n* = 12; mean age 50.8 ± 11.3 years; 51.5 ± 10.1 years	Fenugreek seeds in capsule	Fenugreek (2 g/day); 3 capsules/day after meal for 12 weeks; followed up before initiation of the regimen and then after 12 weeks	5 mg Glibenclamide/day	Fenugreek group (*n* = 6); Glibenclamide group (*n* = 6)	Examined FBG, HbA1c, HOMA‐IR, HDL, LDL, TG, TC, HDL/LDL ratio	Najdi et al. (2019)
Single‐blind, RCTs on diabetic people with prediabetics	*n* = 140; age 30–70 years	Defatted fenugreek powder	Fenugreek powder 5 g before 1/2 h of meal twice a day; followed once in 3 months up to 3 years	Undefined	Control (*n* = 66); study (*n* = 74)	Examined weight, BMI, waist‐to‐hip ratio, FPG, PPG, serum cholesterol, TG, HDLc, LDLc, and serum insulin	Gaddam et al. (2015)
Double‐blind, clinical trial on advanced type 2 DM patients	*n* = 76; age 25–70 years, FBS > 130 mg/dL	Herbal medicine where fenugreek seed was 20% (w/w)	Herbal medicine; one capsule three times a day; 12‐week treatment program	Placebo (micro‐crystalline cellulose 50% and bran 50%); one capsule three times a day	Case (*n* = 38); control (*n* = 38)	Examined serum glucose, urea, creatinine, triglycerides, cholesterol, ALT, AST, ALP, CRP, LDL, HOMA‐IR	Parham et al. (2020)
Clinical trial on 24 type 2 DM patients	*N* = 24; age > 30 years	Powdered fenugreek seeds	Group A (5 g fenugreek seeds powder in 250 g yogurt); Group B (5 g fenugreek seed powder in one quart of hot water); 2 times/day for 2 months	Undefined	Group A (*n* = 12) and Group B (*n* = 12)	Investigated weight, FBS, HbA1c, total cholesterol, LDL, HDL, and food records were measured before and after the study	Kassaian et al. (2009)
Double‐blind, placebo‐controlled study on type 2 DM patients	*n* = 25; M = 19, F = 6; mean age 9.16 ± 6.57 years; 52.83 ± 8.3 years; FBG >200 mg/dL	Hydroalcoholic extract of fenugreek seeds	Hydroalcoholic extract (1 g/day) of fenugreek seeds in capsule; 2 capsules/day before meals for 2 months	Usual care (Dietary control, exercise) and placebo capsule	Group I (*n* = 12; M = 11, F = 1); Group II (*n* = 13, M 8, F = 5)	Analyzed OGTT, lipid levels, fasting C‐peptide, HbA1c, HOMA‐IR	Gupta et al. (2001)
Single‐blinded, RCTs on 62 type 2 DM patients	*n* = 60; M = 45, F = 15; mean age 48 ± 16.25, 46.22 ± 12.25 years; FBG 154.22 ± 30.11, 160.11 ± 28.11	Fenugreek seeds soaked in hot water	10 g/day fenugreek seeds soaked in water before meals; followed up every month for 6 months	Antidiabetic medications	Group A (*n* = 30, M = 21, F = 9); Group B (*n* = 30, M = 24, F = 6)	Parameters assessed were FBG, HbA1c	Ranade and Mudgalkar (2017)
Randomized, double‐blind clinical trial on type 2 DM patients	*n* = 8; age 18–70 years	Fenugreek bread contains 2.5 g of fenugreek seeds	Two slices of bread/day	Wheat bread	Undefined	Assessed blood glucose and insulin levels	Losso et al. (2009)
Double‐blind, randomized, placebo‐control on type 2 DM patients	*n* = 154; M = 108; F = 46; age 25–60 years; FBG ≤180 mg/dL	1000 mg/day Fenugreek seed extract (Fenfuro™) contains 40% furostanolic saponins	Two capsules (500 mg)/day; evaluated over a period of 90 days	Placebo capsule	Treatment group (M = 63.6%, F = 36.4%); Placebo group; (M = 76.6%, F = 23.4%)	Assessed body weight, blood pressure, and pulse rate, fasting and postprandial plasma sugar, HbA1c, fasting and postprandial C‐peptide levels	Verma et al. (2016)
Double‐blind, randomized, placebo‐control study on type 2 DM patients	*n* = 204; average age, 52.22 years; HbA1c >7.5%	1 g/day fenugreek seed extract contains furostanolic saponins	Two 500 mg capsules/day; evaluated after 12 weeks	Placebo	204 patients; Treatment group, (M: F, 68.5: 31.5); Placebo (M: F, 67.7: 32.3)	FPG, PPG, HBA1c, insulin resistance by HOMA‐IR, postprandial C‐peptide, and insulin levels were assessed	Hota et al. (2023)
Double‐blind, randomized study on type 2 DM	*n* = 50; HbA1c > 7.0%; FBG > 7.0 mmol/L	Berberine (300 mg) and fenugreek seed (200 mg)	Three 500 mg capsules/day; assessed after 12 weeks	Placebo	50 patients 25 in each group	Anthropometric measurements and biochemical measurements such as FBS, insulin resistance, sensitivity, beta cell function, C‐reactive protein, TC, TG, LDL, and HDL were evaluated	Nematollahi et al. (2022)
Type 2 DM patients with dyslipidemia	*n* = 50; age 40–60 years; FBG ≥126 mg/dL	Fenugreek seed powder	5 g four times/ day; evaluated after 8 weeks	Oral hypoglycemic drugs	50 patients in two groups based on age	Assessed FBG, lipid (TC, TG, LDL, HDL), HbA1c	Kumar et al. (2015)
Double‐blind, multiple‐dose, randomized, placebo‐controlled, single‐treatment period pilot study on healthy patients	*n* = 13; M = 6, F = 7; mean age 30.07 ± 10.06 years	*Trigonella foenum‐graecum* in 500 mg hard gelatine capsule	2 capsules (500 mg each); on first day 2 capsules at noon and evening and on last day 2 capsules in evening; evaluated for 10 days	Placebo (filled with grits) in capsule	Treatment (M = 2, F = 4); Placebo (M = 4, F = 3)	Assessed the insulin‐sensitizing effect and tested a hypothesis that MCH acted as a critical determinant of this effect using HEGC study	Kiss et al. (2018)
Random, crossover design on NIDDM patients	Study I (10 days): *n* = 15; M = 10, F = 5; mean age 46 ± 3 years; Study II (20 days): *n* = 5; mean age 42 ± 5 years	Defatted fenugreek seed powder	Diets with fenugreek seeds (100 g) powder during lunch and dinner for 10–12 days	Identical nutrient composition except for fiber.	Study I: *n* = 15 (M = 10, F = 5); Study II: *n* = 5	Performed glucose tolerance test, blood glucose, and insulin levels, serum cholesterol, TG levels	Sharma and Raghuram (1990)
Randomized, crossover study on healthy and NIDDM patients	Acute studies on healthy subjects: fenugreek seeds (Whole: *n* = 8, mean age 35 years; Extracted: *n* = 6 (M = 5, F = 1), mean age 34 years; Gum isolate: *n* = 6, mean age 25 years; Degummed: *n* = 6, mean age 32 years; Cooked: *n* = 8, mean age 31 years); Cooked fenugreek leaves: *n* = 4, mean age 30 years; Chronic studies: *n* = 5 NIDDM patients	Seeds, leaves, gum isolate, Acute study: whole, extracted, gum isolate, degummed, cooked seeds or cooked leaves; Chronic study: extracted seeds	Seeds, leaves, gum isolate: (25, 150, 5 g); Whole, extracted, gum isolate, degummed, cooked fenugreek seeds and cooked fenugreek leaves were supplied to healthy volunteers in acute study (single dose); in chronic study, extracted fenugreek seeds were taken for 21 days	Undefined	Acute study: *n* = 38 healthy volunteers; Chronic study: *n* = 5 diabetic volunteers	Assessed plasma glucose and insulin response	Sharma (1986)
Randomized, single‐blind study on 64 T2DM patients	*n* = 64; age 30–60 years	Isolated galactomannan from seeds	1 gm/day Galactomannan in capsule supplied before meal; 4 weeks washout period and performed for 12 weeks	Placebo	Galactomannan group (*n* = 32); Control (*n* = 32)	Assessed FBG, HbA1c, and lipid profile	Rashid et al. (2019)
Type 2 DM patients	*n* = 42; FBG >120 mg/dL, Postprandial blood sugar >140 mg/dL	Fenugreek seeds powder	Powdered fenugreek seeds in Groups I and II, 10 and 20 g/day; assessed at 2‐week interval for 6 weeks	Group III: Normal diet and drug regimen	Group I (*n* = 14); Group II (*n* = 14); Group III (*n* = 14)	Assessed FBG, PPG, HbA1c	Zargar et al. (1992)
Clinical trial on type 2 DM patients	*n* = 108	Fenugreek seeds in powder form	Groups A, B, C; 5, 10, and 15 g/day, respectively; assessed after 2‐week interval 1 month	Undefined	3 Groups: A, B & C	Assessed anthropometric data, GTT, blood glucose, and serum lipid profile	Phadnis et al. (2011)
Clinical trial on type 2 DM patients	*n* = 20; age 40–70 years	10 g of fenugreek seeds soaked into 40 mL of water and boiled fenugreek seed extract	Boiled and liquid seed extract 10 g in 40 mL; assessed 15 days, 30 days, 60 days, and 90 days of interval	Undefined	Undefined	Analyzed blood glucose levels	Hasan and Rahman (2016)
Randomized, crossover, metabolic study on NIDDM patients	*n* = 10; age mean 46.6 ± 2 years	Powdered fenugreek seed	25‐g fenugreek divided and incorporated in chapati during lunch and dinner; assessed after two 15‐day periods	Standard diet without fenugreek	10 patients in two groups	Performed IVGTT, erythrocyte insulin receptor, AUC, half‐life, and metabolic clearance rate analysis	Raghuram et al. (1994)
Clinical trial on type 2 DM patients	*n* = 114; M = 54, F = 41; FBG ≥150 mg/dL; abnormal lipid profile	Seed powder solution	25‐g seed powder in solution twice a day; follow‐up for 1 month	Metformin	Treatment (*n* = 57, M = 27, F = 22); Control (*n* = 57, M = 27, F = 19)	Assessed TC, TG, HDLc, LDL‐C levels	Geberemeskel et al. (2019)
A pilot study on NIDDM patients	*n* = 60; M = 60	Powder mixture (bitter gourd, jamun seeds, and fenugreek seeds); Fenugreek seeds were prepared after overnight soaking, drying, and further grinding	Groups I and II: raw powder mixture in capsule; salty biscuits; 1 g/day for 1.5 months and further 2 g/day for 1.5 months along with lunch and dinner	Undefined	Group I (*n* = 30, M = 30); Group II (*n* = 30, M = 30)	Analyzed serum for glucose	Kochhar and Nagi (2005)
Randomized, double‐blind clinical trial on type 2 DM	*n* = 62, M = 50%, F = 50%; age 3570 years; BMI <35 kg/m^2^	Fenugreek seed powder	10 g/day fenugreek seed powder before meals; assessed after 2 months	Placebo group (10 g of wheat flour/day)	Fenugreek (*n* = 31); placebo (*n* = 31)	Assessed FBG, HbA1c, BMI, waist circumference, diastolic blood pressure, quality of life	Hassani et al. (2019)
Randomized, single‐blind clinical trial on type 2 DM patients	*n* = 12, M = 6, and F = 6	Indian rennet/paneer dodi flowers and fenugreek seeds	300 g of chickpea pulao (3% fenugreek and Indian rennet) for 7 days	300‐g chickpea pulao without herbs	Treatment = 6; control = 6	Assessed PPG	Arooj et al. (2024)

Fenugreek seed supplementation also delays the onset of diabetes. Gaddam et al. (2015) conducted a study on 140 prediabetic subjects for 3 years by administering them 10 g/kg of fenugreek seed powder. Subjects enrolled in this single‐blind, randomized controlled study had an FPG of 100–125 mg/dL and a BMI of ≥19 kg/m^2^. However, fenugreek supplementation lowered the onset of diabetes in prediabetes 4.2 times compared to the control group. In addition, significant reductions were reported in FPG and postprandial plasma glucose (PPG) levels by 99.7 ± 11.4 mg/dL and 129.0 ± 29.6 mg/dL, respectively, in the fenugreek‐treated group. The fenugreek‐treated group also showed significantly higher serum insulin (*p* < .01) and lower LDL (*p* < .05) levels. This study also highlighted the negative association with insulin resistance with fenugreek. The other parameters, including serum cholesterol, HDL, and TG levels, remained unaltered at the end of the study (Gaddam et al., 2015).

Kiss et al. (2018) assessed the insulin‐sensitizing effect of fenugreek seeds (500 mg) on 13 healthy volunteers for 10 days and tested the hypothesis that melanin‐concentrating hormone (MCH) could be a critical factor in this effect by using a hyperinsulinemic euglycemic glucose clamp (HEGC). Fenugreek seeds increased fasting insulin levels and insulin sensitivity by reducing circulating MCH levels (Kiss et al., 2018). Verma et al. (2016) investigated the effect of fenugreek seed extract enriched with furostanolic saponins (Fenfuro™; 500 mg bid) through a double‐blind, randomized, placebo‐controlled study on 154 type 2 DM subjects for 90 days. The Fenfuro™ with metformin regimen reduced FPG and PPG levels by 21.98% versus 7.6% and (30.04% vs. 17.4%), respectively, compared with placebo. Furthermore, significant reduction of HbA1c and improvement in fasting and postprandial C peptide were also reported in the Fenfuro™ treated group compared with their respective baseline values (Verma et al., 2016). A recent double‐blind study of Fenfuro on 204 type 2 DM patients reported that Fenufuro at a dose of 1 g/day significantly reduced PPG by more than 33%, and also decreased FPG levels compared to sulfonylurea or metformin therapy. In addition, insulin sensitivity increased along with a > 10% decrease of serum insulin. However, this study also described that no adverse effects were noticed throughout the 12‐week study period (Hota et al., 2023).

Fenugreek also increased the quality of life in type 2 DM patients. This study was performed by Hassani et al. (2019) on 62 type 2 DM patients for 2 months and reported that 10 g/day fenugreek seeds significantly suppressed FBG, HbA1c, body mass index (BMI), systolic and diastolic blood pressure, and wrist circumference (Hassani et al., 2019). Another study also reported about the quality of life, where a combination of fenugreek seeds and berberine with a value of 200 and 400 mg, respectively, was used for 12 weeks. Fasting insulin and high‐sensitivity C‐reactive protein levels decreased significantly over the study compared to baseline. In addition, the insulin resistance, FBS, and fasting insulin values were also significant compared to the control group (Nematollahi et al., 2022).

Kumar et al. (2015) conducted a study on 50 type 2 DM patients with dyslipidemia for 8 weeks. Fenugreek seed powder 5 g four times/day before meal decreased FBG, total cholesterol (TC), TG, LDL and increased HDL by 178 ± 72.4 to 104 ± 28.2, 350 ± 20.6 to 176 ± 17.2, 280 ± 18.2 to 132 ± 16.8, 220 ± 21.4 to 96 ± 14.2, and 27.0 ± 13.4 to 58 ± 32.2, respectively, compared with their respective initial value. The level of HbA1c was also reduced but the result was not significant (Kumar et al., 2015). Another hyperlipidemic investigation of fenugreek seeds reported that 25‐mg powder solution twice/daily for 1 month reduced TC, TG, and LDL levels by 13.6, 25.53, and 23.4% and increased HDL level significantly by 21.7% compared to control (Geberemeskel et al., 2019). Apart from these, Hasan and Rahman (2016) reported that 10 g of fenugreek soaked either in 40 mL of boiled or unboiled water reduced blood glucose levels. This study was conducted on 20 type 2 DM patients for 90 days (Hasan & Rahman, 2016). In an earlier study conducted by Phadnis et al. (2011) on 108 type 2 DM patients also described that 10 g/day of fenugreek seeds reduced FBG and PPG levels whereas 15 g/day mildly reduced blood glucose level. The seed powder was given at 5, 10, and 15 g/day for 1 month (Phadnis et al., 2011). Arooj et al. (2024) recently conducted a clinical trial on 12 patients by administering 300 g of chickpea pulao. The 3% boiled fenugreek and Indian rennet were used as a broth medium to make the chickpea pulao, and it significantly reduced PPG compared to the control (Arooj et al., 2024).

A fenugreek polysaccharide named galactomannan was also used in the management of hyperglycemia and dyslipidemia. A randomized, single‐blind study of galactomannan was investigated recently by Rashid et al. (2019) on 64 newly diagnosed type 2 DM patients for 12 weeks. At the end of 12 weeks, galactomannan at 1 g/day significantly reduced FBG and HbA1c in comparison with control by 6.30 ± 0.44 versus 7.47 ± 0.30 and 6.23 ± 0.58 versus 7.18 ± 0.17, respectively. Galactomannan also decreased TG, total blood cholesterol (TBC), and LDL significantly, whereas the effect on HDL was not significant (Rashid et al., 2019). Repin et al. (2017) conducted a study on 15 subjects at risk for type 2 DM to find the mechanism of SDF consumption on PPG and insulinemic and gastric emptying responses. Maltose syrup and starch‐based pudding treatments with yellow mustard mucilage, soluble flaxseed gum, or water extract of fenugreek gum at concentrations to match three times the apparent viscosity were supplied. However, for the attenuation of postprandial glucose levels, delayed gastric emptying time and amylolysis could be responsible (Repin et al., 2017).

Parham et al. (2020) conducted a double‐blind study of a polyherbal formulation containing nettle leaf, berry leaf, fenugreek seed, onion and garlic, cinnamon bark, and walnut leaf powder at 20, 10, 20, 20, and 10%, respectively, on 76 type 2 DM subjects. Along with previous drugs, the polyherbal formulation significantly reduced FBG, PPG, HbA1c, and insulin resistance (Parham et al., 2020). Another earlier polyherbal formulation has been found where a combination of bitter gourd, jamun seeds, and fenugreek seeds at a dose of 2 g/day was used in raw and cooked form. This formulation reduced FBG by 41 and 31%, and PPG was reduced by 37 and 28%, in raw and cooked form, respectively. This study was performed on 60 non‐insulin‐dependent diabetes mellites (NIDDM) patients with 1 g/day raw powder for 1.5 months and then 2 g/day for another 1.5 months (Kochhar & Nagi, 2005).

We also found several very earlier clinical trials of fenugreek. Gupta et al. (2001) reported that hydroalcoholic extract of fenugreek seeds at 1 g/day significantly decreased FBG, PPG, and HbA1c levels from 148 ± 44 to 119.9 ± 25, 210.6 ± 79 to 181.1 ± 69 mg/dL, and 8.25 ± 1.2 to 7.54 ± 0.9%, respectively. In addition, insulin resistance decreased (86.3 ± 32 vs. 70.1 ± 52) and insulin sensitivity elevated significantly (112.9 ± 67 vs. 92.2 ± 57) in comparison with the placebo group. Furthermore, serum TG also decreased, and HDL levels also increased after fenugreek treatment. This double‐blind, placebo‐controlled study was performed for 2 months on 25 type 2 DM patients (Gupta et al., 2001). Furthermore, breads containing 2.5 g of fenugreek seeds reduced insulin resistance in a randomized, double‐blind study on eight diet‐controlled type 2 DM subjects (Losso et al., 2009). Raghuram et al. (1994) conducted a study of fenugreek seeds on intravenous glucose disposition on 10 NIDDM for 15 days. These researchers reported that 25 g of fenugreek seeds significantly reduced plasma glucose AUC, half‐life, and increased metabolic clearance rate, as well as erythrocyte insulin receptor. This study mentioned that the hypoglycemic effect of fenugreek seeds was associated with acting on insulin receptors at the gastrointestinal level (Raghuram et al., 1994). Sharma and Raghuram (1990) found that defatted fenugreek seed powder at 100 g with diet significantly suppressed FBG, serum cholesterol, LDL, VLDL, and TG levels. Fenugreek also reduced urinary excretion of glucose by 64%–74% in the 10–20 days of the experiment. This study was performed on 15 NIDDM patients for 15 days and on five patients for an additional 20 days (Sharma & Raghuram, 1990). Similar results were also reported in another study, where 10 g and 20 g/day of fenugreek seed powder were used on 42 type 2 DM patients. A larger dose of 20 g/day produced a significant reduction in FBG. However, HbA1c levels were also reduced, but the results were not statistically significant (Zargar et al., 1992). Sharma (1986) evaluated blood glucose and serum insulin response after fenugreek treatment in an acute study (single dose) on 38 healthy subjects and a chronic study on five NIDDM patients for 21 days. In the acute study, (25 g of seeds, 5 g of gum isolate, and 150 g of leaves) either whole fenugreek seeds, extracted fenugreek seeds, gum isolate, degummed fenugreek seeds, cooked fenugreek seeds, or cooked fenugreek leaves were used. In the chronic study, 25 g of fenugreek seeds was used. The whole seeds showed the greatest reduction in glucose area under the curve (AUC) by 42.4%, followed by gum isolate, extracted seeds, and cooked seeds with a value of 37.5, 36.9, and 35.1%, respectively. In addition, insulin response significantly improved, except for degummed seeds and leaves. In the chronic study, both plasma glucose and insulin responses improved (Sharma, 1986).

## 4 CONCLUSION

Diabetes is a complex metabolic disorder that affects millions of people throughout the world. Due to its progressive nature, it leads to micro‐ and macrovascular complications over time. The 98 preclinical investigations suggested numerous potential compounds present in the fenugreek, including galactomannan, 4‐HIL, GII, trigonelline, sapogenin, diosgenin, quercetin, N55, rhaponticin, vitexin, isovitexin, orientin, and isoorientin. Several pathways were highlighted for the antidiabetic activity of fenugreek and its isolated compounds. They activated the PI3K/Akt pathway, which regulated IRS‐1, Akt, and GSK‐3 phosphorylation and GLUT4 translocation to reduce hyperglycemia. Other highlighted mechanisms were inhibition of carbohydrate metabolizing enzymes, restoration of glucose utilizing and metabolizing enzymes, protection of pancreatic β cells, and improvement of insulin sensitivity. Furthermore, fenugreek and isolated compounds also activated AMPK and GLP‐1, inhibited DPP‐IV inhibition, and prevented oxidative damage. Clinical studies also demonstrated that its seeds, alone or in combination, are effective in lowering hyperglycemia and insulin resistance. Overall, we found strong evidence of the antidiabetic effect of fenugreek; nevertheless, the underlying molecular mechanism of fenugreek extracts or isolated components needs more investigation. Furthermore, the optimal dose and treatment duration in clinical trials must be established in order to get a beneficial outcome from fenugreek.

## AUTHOR CONTRIBUTIONS

**Dipto Kumer Sarker:** Conceptualization (lead); data curation (lead); formal analysis (lead); investigation (lead); methodology (lead); resources (lead); software (lead); validation (lead); visualization (lead); writing – original draft (lead). **Pallobi Ray:** Data curation (equal); formal analysis (equal); investigation (equal); methodology (equal); software (equal); writing – original draft (equal). **Ashit Kumar Dutta:** Investigation (supporting); methodology (supporting). **Razina Rouf:** Writing – review and editing (equal). **Shaikh Jamal Uddin:** Conceptualization (lead); project administration (lead).

## CONFLICT OF INTEREST STATEMENT

The authors declare no conflict of interest regarding this paper.

## Data Availability

All data used to reach the conclusion are freely available in the manuscript.
